# Secondary metabolic profiling of *Serratia marcescens* NP10 reveals new stephensiolides and glucosamine derivatives with bacterial membrane activity

**DOI:** 10.1038/s41598-023-28502-6

**Published:** 2023-02-09

**Authors:** Tanya Clements-Decker, Marina Rautenbach, Wilma van Rensburg, Sehaam Khan, Marietjie Stander, Wesaal Khan

**Affiliations:** 1 Faculty of Health Sciences, University of Johannesburg, P.O. Box 17011, Doornfontein, 2028 South Africa; 2 Department of Biochemistry, Faculty of Science, Stellenbosch University, Private Bag X1, Stellenbosch, 7602 South Africa; 3 Department of Microbiology, Faculty of Science, Stellenbosch University, Private Bag X1, Stellenbosch, 7602 South Africa

**Keywords:** Biochemistry, Microbiology

## Abstract

Secondary metabolic profiling, using UPLC-MS^E^ and molecular networking, revealed the secondary metabolites produced by *Serratia marcescens* NP10. The NP10 strain co-produced cyclic and open-ring stephensiolides (i.e., fatty acyl chain linked to Thr–Ser–Ser–Ile/Leu–Ile/Leu/Val) and glucosamine derivatives (i.e., fatty acyl chain linked to Val–glucose–butyric/oxo-hexanoic acid), with the structures of sixteen new stephensiolides (L–Y) and three new glucosamine derivatives (L–N) proposed. Genome mining identified *sphA* (stephensiolides) and *gcd* (glucosamine derivatives) gene clusters within *Serratia* genomes available on NBCI using antiSMASH, revealing specificity scores of the adenylation-domains within each module that corroborates MS^E^ data. Of the nine RP-HPLC fractions, two stephensiolides and two glucosamine derivatives exhibited activity against *Staphylococcus* *aureus* (IC_50_ of 25–79 µg/mL). ^1^H NMR analysis confirmed the structure of the four active compounds as stephensiolide K, a novel analogue stephensiolide U, and glucosamine derivatives A and C. Stephensiolides K and U were found to cause membrane depolarisation and affect the membrane permeability of *S. aureus*, while glucosamine derivatives A and C primarily caused membrane depolarisation. New members of the stephensiolide and glucosamine derivative families were thus identified, and results obtained shed light on their antibacterial properties and mode of membrane activity.

## Introduction

Many classes of chemotherapeutic agents are secondary metabolites produced by diverse microbial genera, such as *Streptomyces*, *Actinobacteria*, *Bacillus*, *Myxobacteria* and *Serratia,* and include daptomycin, staurosporine, iturin W, myxopyronin and serrawettin W2 analogues^1–5^. However, after the cyclic lipopeptide daptomycin received United States Food and Drug Administration approval as an antibiotic^1^, lipopeptides, such as those produced by *Serratia* species^4,6–8^, became an important drug discovery focus.

Lipopeptides are small molecules comprised of a peptide moiety linked to a fatty acid moiety and vary based on the amino acid composition in the peptide moiety and/or the length and saturation/unsaturation of the fatty acyl chain^9^. Within the *Serratia* genus, serrawettin W1 and W2 (and their respective analogues) have been the focus of lipopeptide research over the past six decades^4,8,10–16^. However, Ganley et al.^17^ recently discovered novel lipopeptides produced by a *Serratia* sp. and described 11 new compounds with their nomenclature as stephensiolides A–K (*m/z* 600 to 696 [M + H]^+^). Stephensiolides are a group of cyclic lipopeptides comprised of one fatty acid chain (varying in saturation and length from C_8_ to C_14_) and a cyclised amino acid moiety of Thr-Ser-Ser-Val/Ile-Val/Ile, with Thr (first amino acid) linked to Val/Ile (fifth amino acid) via an ester bond, and to Ser (second amino acid) through an amide bond^17^. The potential bioactivity of a mixture of the stephensiolides was subsequently investigated, and was found to exhibit activity against *Bacillus subtilis* 3610 and chloroquine-resistant *Plasmodium falciparum* Dd2 blood-stage parasites. Thereafter, Mai et al.^18^ isolated a *Lecanicillium* sp. strain (an endophyte associated with *Sandwithia guyanensis*) capable of producing stephensiolides. Five of the identified stephensiolides (analogues to be abbreviated as Stp followed by the allocated letter), StpC, StpD, StpF, StpG and StpI, were purified from the extract; four of which (StpD, StpF, StpG and StpI) displayed promising activity against methicillin-resistant *Staphylococcus aureus* (MRSA) with minimum inhibitory concentrations (MICs) of 4–128 µg/mL.

The co-production of lipopeptides (i.e., serrawettin W1) with glucosamine derivatives by certain *Serratia* strains, has also been described^8,14^. Glucosamine derivatives are small molecules (*m/z* 545 to 627 [M + H]^+^) comprised of a sugar (glucose/hexose), amino acid (valine), fatty acid (C_13_ to C_17_) and butyric or oxo-hexanoic acid^8,14^. Glucosamine derivative analogues (to be abbreviated as Glu followed by the allocated letter), GluA-GluC and GluE, have been reported to exhibit antibacterial activity against *Mycobacterium* spp. and *Enterococcus faecalis*^8,14^. However, for both the stephensiolides and the glucosamine derivatives, limited information is currently available on the mechanism of action employed by these compounds to target microbial species.

The aim of this study was thus to describe the structures of new stephensiolide and glucosamine derivative analogues co-produced by a non-pigmented *S. marcescens* NP10 strain, which was isolated from river water^7^. A combined approach of ultra-performance liquid chromatography coupled to tandem mass spectrometry (UPLC-MS^E^), molecular networking using Global Natural Product Social Molecular Networking (GNPS; https://gnps.ucsd.edu/), ^1^H NMR (conducted on four purified active compounds) and genome mining using sequences available on National Center for Biotechnology Information (NCBI; https://www.ncbi.nlm.nih.gov/) combined with the "antibiotics and secondary metabolite analysis shell—antiSMASH" (https://antismash.secondarymetabolites.org/), was utilised to identify and elucidate the new stephensiolides and glucosamine derivatives produced by the NP10 strain. A secondary aim involved elucidating the antibacterial, membrane disruption and membrane depolarisation activities of stephensiolides and glucosamine derivatives fractions, purified from the extract via reverse-phase high-performance liquid chromatography (RP-HPLC).

## Results

### Preliminary identification: UPLC-MS analysis of the *S. marcescens* NP10 extract

Following UPLC-MS^E^, the high-resolution (HR) UPLC-MS analysis (MS1 analysis) revealed 28 primary compounds (abundance of 0.2–7.2%) within the NP10 extract eluting between 4.95 and 11.88 min [Table 1 (stephensiolides) and Supplementary Table S1 (glucosamine derivatives)]. Comparison to literature and online databases revealed that nine of the 28 compounds (i.e., *m/z* 614.3760, 628.3920, 642.4078, 668.4225, 656.4233, 682.4377, 670.4399, 696.4548 and 684.4534 [M + H]^+^) were putatively identified as members of the lipopeptide family, stephensiolides, based on the experimental protonated *M*_*r*_ and elementary composition calculated from the HRMS data (Table 1 and Supplementary Table S2)^17^. Moreover, eight of the 28 compounds (i.e., *m/z* 575.3909, 557.3804, 559.3973, 573.4144, 583.3960, 585.4125, 627.4238 and 587.4270 [M + H]^+^) were putatively identified as members of the glucosamine derivative family, based on the experimental protonated *M*_*r*_ and elementary composition calculated from the HRMS data (Supplementary Tables S1 and S2)^8,14^. The remaining 11 compounds did not correspond to compounds reported in literature and online databases. However, all 28 primary compounds formed sodium- and potassium-adducts and formed stable dimers based on the UPLC-MS analysis (Supplementary Table S2).

**Table 1:** Summary of the chemical parameters and proposed structure of stephensiolide lipopeptides in this study. Sequences in normal font are proposed from the accurate *M*_*r*_, elemental composition and partial sequencing with UPLC-MS^E^. Sequences in bold font were elucidated/confirmed with ^1^H NMR.

No.	R_t_ (min)	Abundance^a^	Experimental *m/z* ^b^ [M + H]^+^	Theoretical *m/z* ^c^ [M + H]^+^	PPM error ^d^	Proposed elementary composition ^e^	Proposed residue in stephensiolides, open-ring or ring closure with ester between residue 1 and 6						Original/New name; References
							1	2	3	4	5	6	
1	5.46	0.6	644.3851	644.3871	3	C_30_H_54_N_5_O_10_	C_8_H_15_O_3_	Thr	Ser	Ser	Ile/Leu	Ile/Leu	Stephensiolide L; N/A
2	6.45	3.2	614.3760	614.3765	1	C_29_H_51_N_5_O_9_	C_8_H_14_O_2_	Thr	Ser	Ser	Ile/Leu	Val	Stephensiolide M; N/A
3	7.17	4.7	628.3920	628.3920	0	C_30_H_53_N_5_O_9_	C_8_H_14_O_2_	Thr	Ser	Ser	Ile/Leu	Ile/Leu	Stephensiolide N; N/A
4	4.95	0.4	690.4240	690.4290	5	C_32_H_60_N_5_O_11_	C_10_H_19_O_3_	Thr	Ser	Ser	Ile/Leu	Ile/Leu + OH	Open-ring stephensiolide P; N/A
5	6.22	1.0	674.4318	674.4341	1	C_32_H_60_N_5_O_10_	C_10_H_18_O_2_	Thr	Ser	Ser	Ile/Leu	Ile/Leu + OH	Open-ring stephensiolide R; N/A
6	6.28	4.1	658.4034	658.4034	0	C_31_H_56_N_5_O_10_	C_10_H_19_O_3_	Thr	Ser	Ser	Ile/Leu	Val	Stephensiolide O; N/A
7	7.00	6.7	672.4190	672.4190	1	C_32_H_58_N_5_O_10_	C_10_H_19_O_3_	Thr	Ser	Ser	Ile/Leu	Ile/Leu	Stephensiolide P; N/A
8	8.09	3.5	642.4078	642.4078	0	C_31_H_55_N_5_O_9_	C_10_H_18_O_2_	Thr	Ser	Ser	Ile/Leu	Val	Stephensiolide Q; N/A
9	8.79	4.5	656.4233	656.4234	0	C_32_H_57_N_5_O_9_	C_10_H_18_O_2_	Thr	Ser	Ser	Ile/Leu	Ile/Leu	Stephensiolide R; N/A
10	8.01	1.5	698.4340	698.4341	0	C_34_H_60_N_5_O_10_	C_12_H_23_O_3_	Thr	Ser	Ser	Ile/Leu	Ile/Leu	Stephensiolide S; N/A
11	8.92	4.5	668.4225	668.4234	1	C_33_H_57_N_5_O_9_	C_12_H_20_O_2_	Thr	Ser	Ser	Ile/Leu	Val	Stephensiolide T; N/A
12	9.57	5.2	682.4377	682.4391	2	C_34_H_59_N_5_O_9_	C_12_H_20_O_2_	**Thr**	**Ser**	**Ser**	**Ile**	**Ile**	Stephensiolide U; N/A
13	9.74	3.2	670.4399	670.4391	1	C_33_H_59_N_5_O_9_	C_12_H_22_O_2_	Thr	Ser	Ser	Ile/Leu	Val	Stephensiolide V; N/A
14	10.37	5.9	684.4534	684.4547	2	C_34_H_61_N_5_O_9_	C_12_H_22_O_2_	**Thr**	**Ser**	**Ser**	**Ile**	**Ile**	Stephensiolide K;^17^
15	10.31	0.4	696.4548	696.4547	0	C_35_H_61_N_5_O_9_	C_14_H_24_O_2_	Thr	Ser	Ser	Ile/Leu	Val	Stephensiolide W; N/A
16	10.87	1.4	710.4691	710.4704	2	C_36_H_64_N_5_O_9_	C_14_H_24_O_2_	Thr	Ser	Ser	Ile/Leu	Ile/Leu	Stephensiolide X; N/A
17	11.88	0.5	712.4857	712.4860	0	C_36_H_66_N_5_O_9_	C_14_H_26_O_2_	Thr	Ser	Ser	Ile/Leu	Ile/Leu	Stephensiolide Y; N/A

N/A—Not applicable; No.—Compound number; R_t_—Retention time obtained with UPLC-MS within the NP10 extract.

^a^Abundance was calculated from the UPLC-MS analyses of the crude extract. ^b^Experimental protonated *M*_*r*_ of a compound in the selected extract was calculated using the Time-of-Flight (TOF) transform function in the MassLynx 4.2 software package. ^c^Theoretical protonated *M*_*r*_ of compound was calculated using de novo* M*_*r*_ calculation and verification with ChemDraw Ultra 12.0 software package. ^d^Mass error in ppm = $\left( {\frac{{{\text{Theoretical}}\ M_{r} { } - {\text{Experimental}}\ M_{r} }}{{{\text{ Theoretical}}\ M_{r} }}} \right) \times 10^{6}$. ^e^Theoretical molecular formula of compound was calculated using ChemDraw Ultra 12.0 software package and experimental molecular formula was confirmed using the MassLynx 4.2 software package. Refer to supplementary data for the UPLC-MS^E^ sequence analyses.

### Molecular Networking

**Figure 1:**
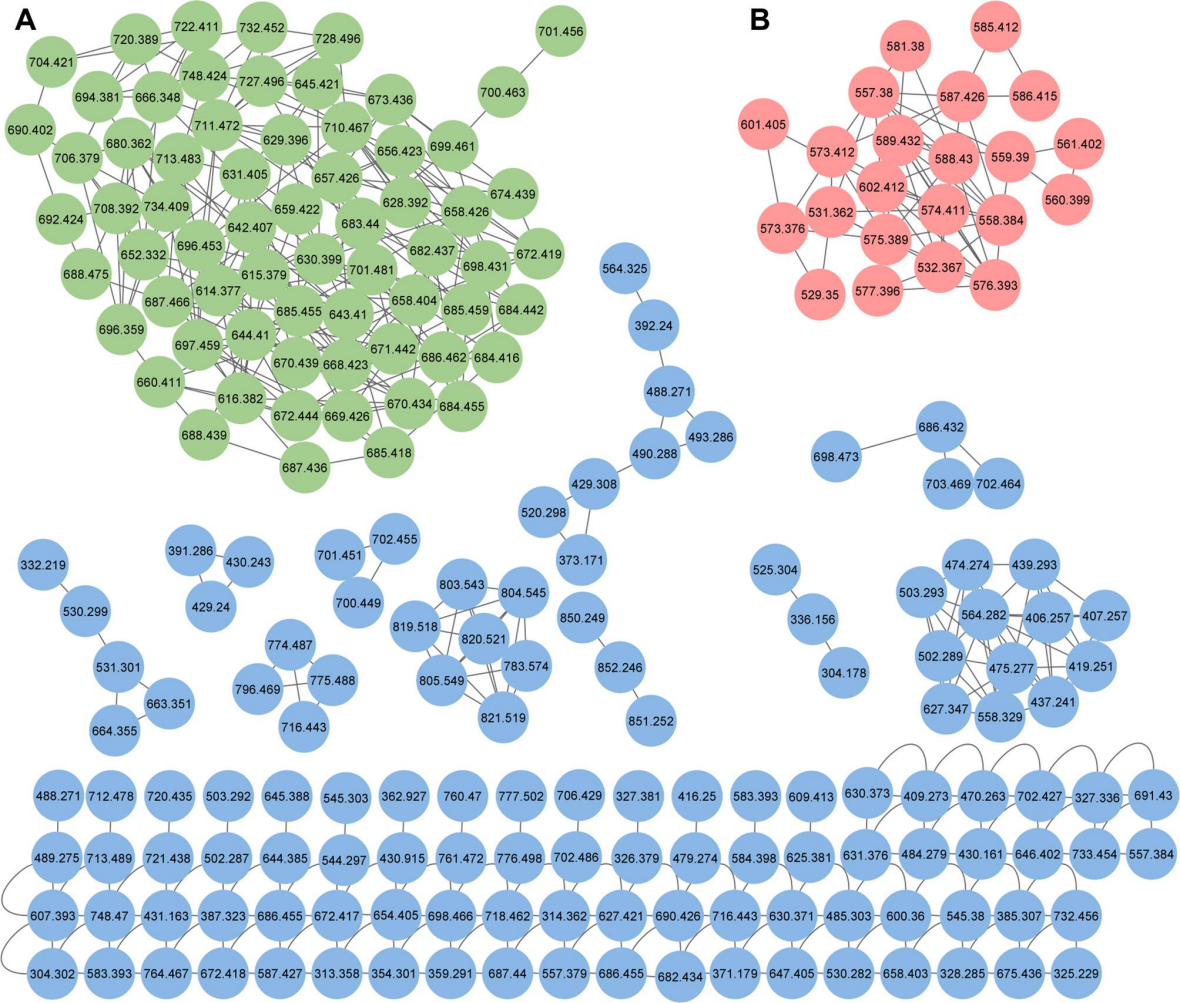
The molecular network was generated on the GNPS platform (http://gnps.ucsd.edu) using the UPLC-MS^E^ data and visualised using Cytoscape version 3.8.0. The green nodes correspond to cluster A (stephensiolides), and the red nodes correspond to cluster B (glucosamine derivatives). The blue nodes correspond to unknown or individual clusters.

Following the preliminary identification of the 28 compounds in the extract, using HRMS data, the MS^E^ data was used to generate a molecular network on the GNPS website. The entire network was comprised of 218 nodes, with two primary clusters (A with 66 nodes and B with 22 nodes) identified (Fig. 1). This suggests that the compounds within each cluster may share similar structures. The nine compounds that were putatively identified as members of the stephensiolide family corresponded to nodes in cluster A (i.e., *m/z* 614.377, 628.392, 642.407, 656.423, 670.434, 684.455, 668.423, 682.437 and 696.453 [M + H]^+^), while seven unknown compounds also corresponded to nodes in cluster A (i.e., *m/z* 644.410, 690.402, 674.439, 658.404, 672.444, 698.431 and 710.467). This implies that cluster A may be comprised of known and unknown stephensiolides (Fig. 1). In addition, six of the eight compounds putatively identified as members of the glucosamine derivative family corresponded to nodes in cluster B (i.e., *m/z* 575.389, 557.38, 559.39, 573.412, 585.412 and 587.426 [M + H]^+^), while three unknown compounds also corresponded to nodes in cluster B (i.e., *m/z* 529.35, 531.362 and 601.405) suggesting that this cluster may also be comprised of known and unknown glucosamine derivatives (Fig. 1). The remaining three compounds (*m/z* 712.478, 583.393 and 627.421 [M + H]^+^), of the 28 detected compounds, did not cluster within either A or B, but were rather detected as individual nodes and structures were subsequently elucidated using CID and MS^E^ analysis.

### Structural elucidation: CID, MS^E^ and ^1^H NMR analysis

#### Stephensiolide family

**Figure 2:**
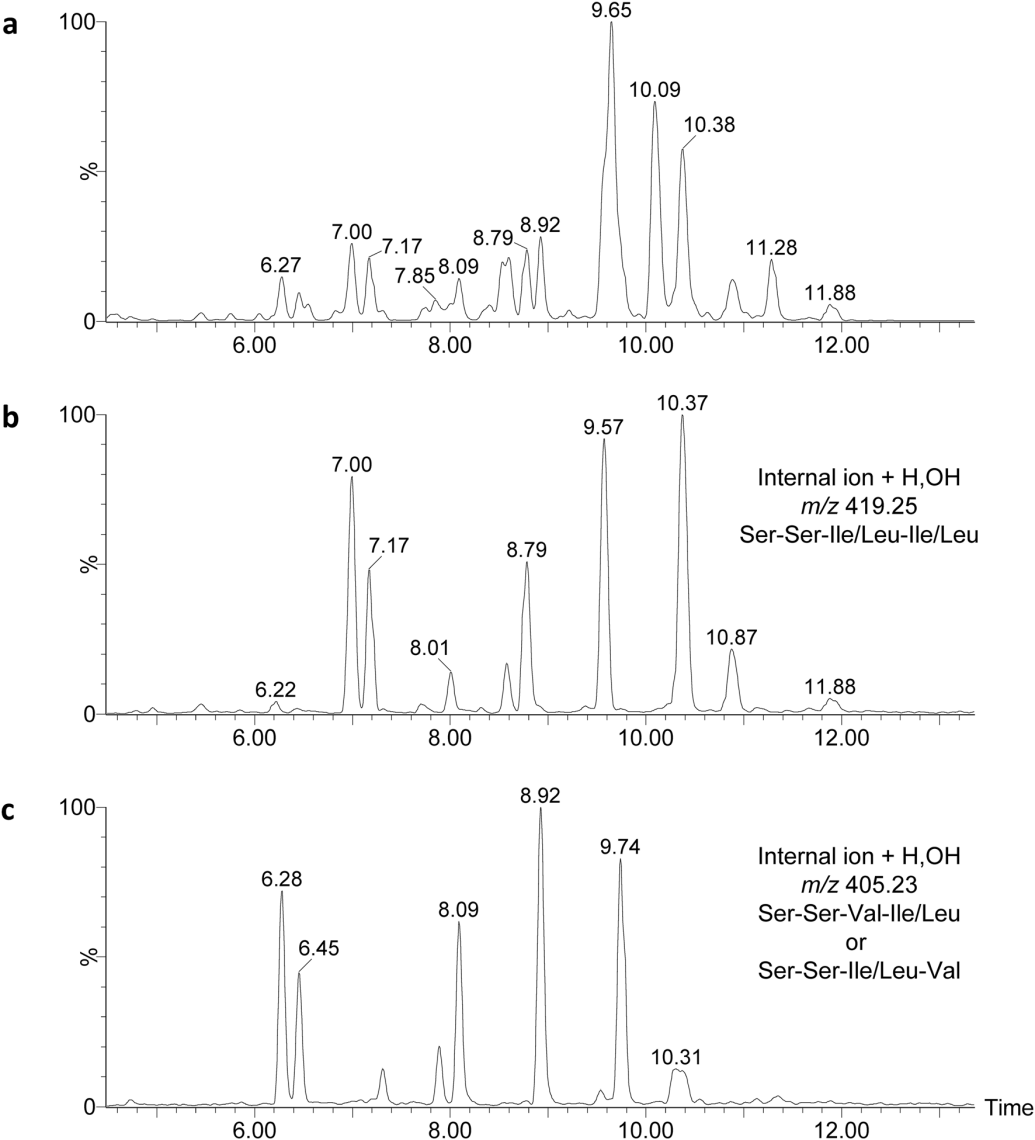
The positive ion chromatogram profile (between 5 and 12 min) of (**a**) total ions in the crude extract, (**b**) ion peaks that have the fragment corresponding to an internal ion (+ H, OH) of Ser-Ser-Ile/Leu-Ile/Leu, and (**c**) ion peaks that have the fragment corresponding to an internal ion (+ H, OH) of Ser-Ser-Ile/Leu-Val or Ser-Ser-Val-Ile/Leu.

As HRMS and molecular networking putatively identified members of the stephensiolide family, internal fragments from CID in the MS^E^ mode could be used to identify related compounds with correlating sequences within the NP10 extract. The structures of stephensiolides were previously described to have five amino acid residues (Thr-Ser-Ser-Val/Ile-Val/Ile) linked to a β-hydroxy fatty acyl residue (C_8_–C_14_)^17^. Therefore, CID fragments generated from the internal ions + H, OH, including *m/z* 419.25 for a Ser-Ser-Ile/Leu-Ile/Leu + H, OH fragment; *m/z* 405.23 for Ser-Ser-Val-Ile/Leu + H, OH or Ser-Ser-Ile/Leu-Val + H, OH fragments; and *m/z* 391.22 for a Ser-Ser-Val-Val + H, OH fragment, were used for preliminary screening of stephensiolide analogues. Based on this, it was determined that the peptides eluting at R_t_ of 6.22, 7.00, 7.17, 8.01, 8.79, 9.57, 10.37, 10.87 and 11.88 min, contained an internal fragment of *m/z* 419.25, indicating that compounds *m/z* 674.4318, 672.4190, 628.3920, 698.4340, 656.4233, 682.4377, 684.4534, 710.4691 and 712.4857 [M + H]^+^ (corresponding to the R_t_, respectively) contained a Ser-Ser-Ile/Leu-Ile/Leu + H, OH fragment (Fig. 2). Moreover, peptides eluting at R_t_ of 6.28, 6.45, 8.09, 8.92, 9.74 and 10.31 contained an internal fragment of *m/z* 405.23, with compounds *m/z* 658.4034, 614.3760, 642.4078, 668.4225, 670.4399 and 696.4548 [M + H]^+^, respectively, containing either a Ser-Ser-Val-Ile/Leu + H, OH or a Ser-Ser-Ile/Leu-Val + H, OH fragment (Fig. 2). There were no peaks observed with an internal fragment of *m/z* 391.22 for Ser-Ser-Val-Val + H, OH (results not shown). The preliminary screening for internal ions indicates that this strain is predominantly producing stephensiolides with an Ile/Leu as residue 5. These stephensiolide analogues are thus distinct from previously described stephensiolides which contain a Val as residue 5 (except for StpK).

**Figure 3:**
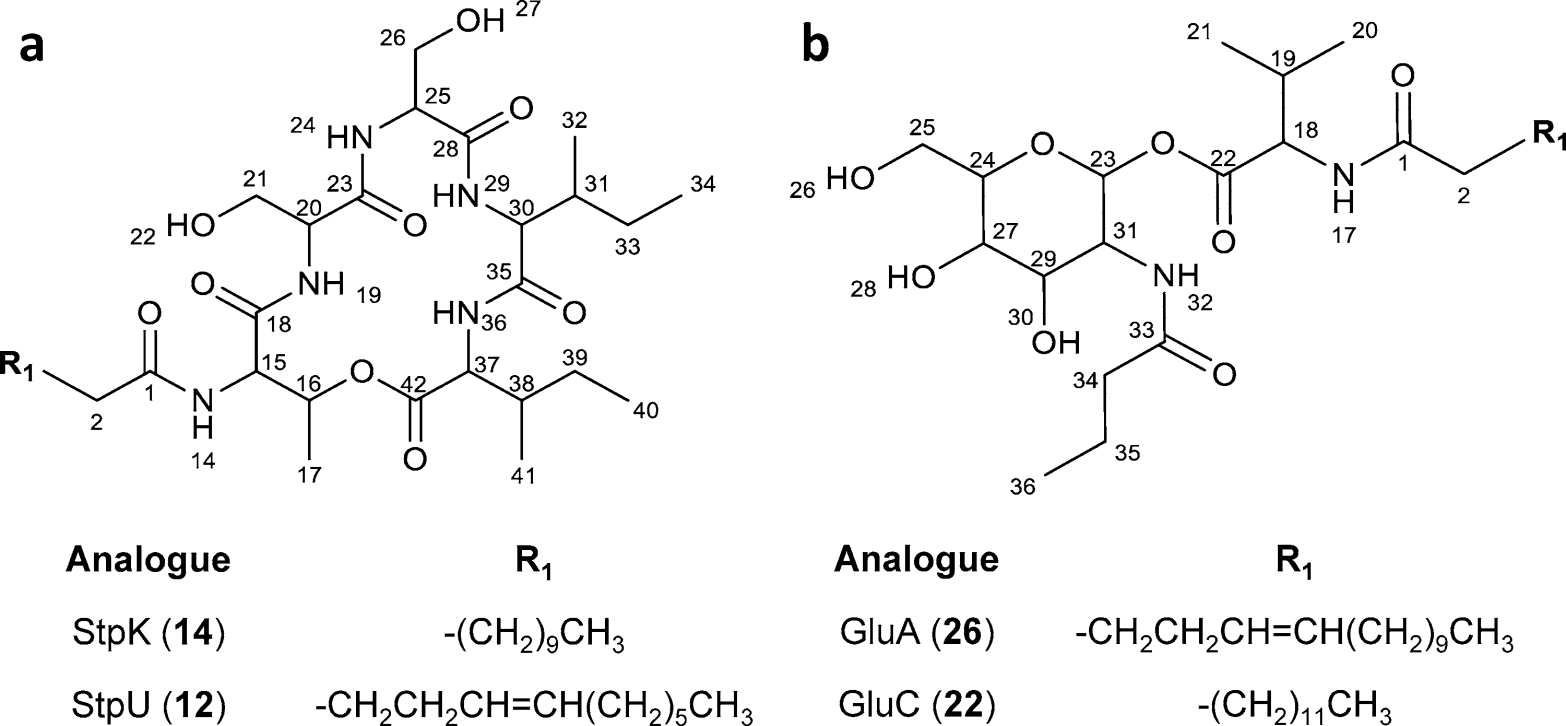
Structures of (**a**) StpK and StpU and (**b**) GluA and GluC.

Fragmentation profile analysis using the UPLC-MS^E^ data was subsequently conducted to elucidate the structures of these new stephensiolide analogues. For most of these lipopeptides, the whole sequence of b-ions (and dehydrated b-ions) were detected in the fragmentation profile and in some cases the corresponding a-ions were also observed (Supplementary Table S3). The CID fragmentation analysis putatively confirmed the structure of *m/z* 684.4534 [C_34_H_61_N_5_O_9_ + H]^+^ (compound **14**) as C_13_H_22_O_2_-Thr-Ser-Ser-Ile/Leu-Ile/Leu (Table 1 and Supplementary Table S3). Furthermore, ^1^H NMR analysis of **14** then confirmed the structure as C_13_H_22_O_2_-Thr-Ser-Ser-Ile-Ile, corresponding to the previously described StpK (Table 2; Fig. 3a and Supplementary Fig. S1)^17,18^. Despite the protonated *M*_*r*_ and elementary composition of compounds *m/z* 614.3760, 628.3920, 642.4078, 656.4233, 668.4225, 682.4377, 670.4399, 684.4534 and 696.4548 [M + H]^+^ (**2**, **3**, **8**, **9**, **11**, **12**, **13**, **14** and **15**, respectively), corresponding to known stephensiolides, distinctive differences were observed in the expected fragmentation pattern (i.e., Ile/Leu at residue 5) (Supplementary Table S3; Supplementary Fig. S2). For example, the analysis of HRMS for compound **12** provided a [M + H]^+^ ion at *m/z* 682.4377, that correlated to the molecular formula C_34_H_59_N_5_O_9_ (Table 1), which was analogous to StpH. However, the MS^E^ fragmentation profile of **12** revealed major product ions at *m/z* 664.428, 551.345, 438.259, 351.231 and 264.197 [M + H]^+^ (Supplementary Table S3) and an internal ion of *m/z* 419.25 corresponding to Ser-Ser-Ile/Leu-Ile/Leu + H, OH (Fig. 2). This putatively directed the identification of structure **12** as C_12_H_20_O_2_-Thr-Ser-Ser-Ile/Leu-Ile/Leu (Table 1) with the initial fragmentation occurring at the ester bond between residue 2 (Thr) and 6 (Ile/Leu). Moreover, ^1^H NMR putatively elucidated the structure of this novel analogue as C_12_H_20_O_2_-Thr-Ser-Ser-Ile-Ile (Table 2; Fig. 3a and Supplementary Figure S1). This compound thus differed from that of the previously described StpH with an identical *m/z* 682.4385 [M + H]^+^ ion and molecular formula of C_34_H_59_N_5_O_9_, with differing residues 1, 5 and 6 (Table S4)^17^. Similar distinctions were observed in the internal ions and MS^E^ analysis for compounds **2**, **3**, **8**, **9**, **11**, **13** and **15**, indicating that these compounds are new stephensiolide analogues and were thus named accordingly (Fig. 2; Table 1, Supplementary Tables S3 and S4).

**Table 2:** The ^1^H NMR Data (600 MHz) for StpK and StpU (in CD_3_CN), as well as GluA and GluC (in CD_3_OD). Refer to Fig. 3 for the atom numbered structures.

**Position (**^**1**^**H)**	StpK	StpU	**Position (**^**1**^**H)**	GluA	GluC
	δ_H_, m (*J* in Hz)	δ_H_, m (*J* in Hz)		δ_H_, m (*J* in Hz)	δ_H_, m (*J* in Hz)
**Lipid**	**Fatty acid**	**Fatty acid**	**Lipid**	**Fatty acid**	**Fatty acid**
2	1.84, m	1.83, m	2	2.03, d	2.21, m
3	1.62, m	1.65, m	3	1.61, m	1.61, m
4	1.28, s	2.05, m	4	2.24,m	1.29, m
5	1.28, s	5.38, m	5	5.34, m	1.29, m
6	1.28, s	5.38, m	6	5.34, m	1.29, m
7	1.28, s	2.05, m	7	2.24, m	1.29, m
8–11	1.28, s	1.28, m	8–13	1.31, m	1.29, m
12	0.89, m	0.89, m	14	1.31, m	0.91, dt
**Residue 1**	**Thr**	**Thr**	15	1.31, m	–
14 (N*H*)	7.64, d	7.60, d	16	0.91, m	–
15	5.33, dd	5.32, dd	**Residue 1**	**Val**	**Val**
16	5.28, dd	5.27, dd	17 (N*H*)	–	–
17	0.95, dd	0.94, dd	18	4.34, d (6.2); 4.32, d (5.9)	4.34, d (6.2); 4.31, d (6.1)
**Residue 2**	**Ser**	**Ser**	19	2.24, m	2.29, dt
19 (N*H*)	7.46, d	7.43, d	20	0.99, m	0.99, m
20	4.55, dd	4.55, dd	21	0.99, m	0.99, m
21a	4.27, m	4.22, m	**Residue 2**	**Glucosamine**	**Glucosamine**
21b	4.18, m	4.14, m	23	6.22, d (3.2); 6.19, d (3.3)	6.22, d (3.2); 6.19, d (3.3)
22 (O*H*)	3.86, dd	3.86, dd	24	3.76, m	3.76, m
**Residue 3**	**Ser**	**Ser**	25a	3.76, m	3.76, m
24 (N*H*)	7.35, d	7.32, d	25b	3.70, m	3.70, m
25	4.55, dd	4.55, dd	26 (O*H*)	3.70, m	3.70, m
26a	4.27, m	4.22, m	27	3.47, m	3.47, dd
26b	4.18, m	4.14, m	28 (O*H*)	3.66, m	3.65, m
27 (O*H*)	3.78, ddd	3.78, ddd	29	4.04, dd (10.9, 3.3); 3.97, dd (10.9; 3.2)	4.04, dd (10.9, 3.3); 3.97, dd (10.9; 3.2)
**Residue 4**	**Ile**	**Ile**	30 (O*H*)	3.66, m	3.65, m
29 (N*H*)	7.02, d	7.00, d	31	4.58, s	4.59, d
30	4.44, m	4.44, m	**Residue 3**	**Butyric acid**	**Butyric acid**
31	1.84, m	1.84, m	32 (N*H*)	–	–
32	0.89, m	0.89, m	34	2.03, d	2.21, m
33	1.62, m	1.65, m	35	1.61, m	1.61, m
34	0.89, m	0.89, m	36	0.91, m	0.91, dt
**Residue 5**	**Ile**	**Ile**			
36 (N*H*)	6.93, d	6.90, d			
37	4.39, m	4.39, m			
38	2.07, m	2.05, m			
39	1.62, m	1.65, m			
40	0.89, m	0.89, m			
41	0.89, m	0.89, m

The preliminary structural elucidation of eight additional new stephensiolides (compounds **1**, **4**–**7**; **10**, **16** and **17**), observed in cluster A of the molecular network, was conducted using internal ions and detailed MS^E^ fragmentation analysis. The analysis of HRMS for compounds **16** and **17** had prominent [M + H]^+^ ions of *m/z* 712.4857 and 710.4691, that supports molecular formulas of C_36_H_64_N_5_O_9_ and C_36_H_66_N_5_O_9_, respectively (Table 1). The MS^E^ analysis then revealed the putative structure of **16** and **17** as C_14_H_24_O_2_-Thr-Ser-Ser-Ile/Leu-Ile/Leu and C_14_H_26_O_2_-Thr-Ser-Ser-Ile/Leu-Ile/Leu, respectively, cyclised between residue 2 (Thr) and 6 (Ile/Leu) (Supplementary Fig. S2). Thus, **16** and **17** were distinct from one another based exclusively on the presence of a double bond in the fatty acyl chain of **16**. Moreover, the HRMS analysis of **1**, **4**, **5**, **6**, **7** and **10** revealed prominent [M + H]^+^ ions of *m/z* 644.3851, 690.4240, 674.4318, 658.4034, 672.4190 and 698.4340 that supports molecular formulas of C_30_H_54_N_5_O_10_, C_32_H_60_N_5_O_11_, C_32_H_60_N_5_O_10_, C_31_H_56_N_5_O_10_, C_32_H_58_N_5_O_10_ and C_34_H_60_N_5_O_10_ (Table 1), respectively, highlighting that these compounds have either one or two additional hydroxyl groups. The MS^E^ fragmentation analysis then putatively confirmed that the structures of **1**, **6**, **7** and **10**, had the additional hydroxyl group within the fatty acid chain, while **4** and **5** were open-ring structures of **7** and **9**, respectively (Table 1 and Supplementary Table S4; Supplementary Fig. S2).

#### Glucosamine derivative family

The molecular networking (cluster B) and detailed MS^E^ fragmentation analysis were used to putatively identify the structures of *m/z* 575.3909 (**20**), 557.3804 (**21**), 559.3973 (**22**), 573.4144 (**23**), 583.3960 (**25**), 585.4125 (**26**), 627.4238 (**27**) and 587.4270 (**28**) [M + H]^+^ as previously described GluD, GluE, GluC, GluB, GluH, GluA, GluJ and GluK, respectively (Supplementary Tables S1 and S5)^8,14^. In addition, ^1^H NMR confirmed the structures of 559.3973 (**22**) and 585.4125 (**26**) (Table 2; Fig. 3b and Supplementary Fig. S1), corresponded to the ^1^H NMR detected by Dwivedi et al.^14^ for GluC and GluA, respectively. In addition, the structures of three new glucosamine derivatives (compounds **18**, **19** and **24**), detected in cluster B of the molecular network, were putatively elucidated using MS^E^ fragmentation analysis. The analysis of HRMS for compounds **18** and **19** provided a prominent ion with *m/z* 529.3513 and 531.3650 [M + H]^+^ that supports a molecular formula of C_27_H_49_N_2_O_8_ and C_27_H_51_N_2_O_8_, respectively (Supplementary Table S1), while analysis of HRMS for compound **24** provided a prominent ion with *m/z* 601.4042 [M + H]^+^ that supports a molecular formula of C_31_H_57_N_2_O_9_ (Supplementary Table S1).

Similar to previously described glucosamine derivatives^8,14^, the cleavage of the three new compounds occurred at the anomeric C-O bond resulting in two major fragments for each compound. This resulted in a major fragment of *m/z* 232.116 for **18**, **19** and **24** corresponding to the glucose/hexose residue linked to butyric acid, and dehydration products of the *m/z* 232.116 fragment were observed at *m/z* 214.107 and 196.097 (Supplementary Table S5, Supplementary Fig. S3). Compounds **18** and **19** had a second major fragment of *m/z* 298.238 and 300.254, which corresponded to Val residue linked to a saturated C_12_ and unsaturated C_12:1_ fatty acyl chain, respectively (Supplementary Table S5, Supplementary Fig. S3). In comparison, compound **24** had a second major fragment of *m/z* 370.295, which corresponded to a Val residue linked to a saturated C_16_ fatty acyl chain with an additional hydroxyl group and double bond in the fatty acyl residue.

### Genome mining, antiSMASH and NRSPpredictor2

The biosynthetic gene cluster (BGC), *sphA*, encoding for hybrid polyketide synthases (PKS) and non-ribosomal peptide synthetases (NRPSs) involved in stephensiolide biosynthesis, was identified in the genomic data of 16 *Serratia* strains available on the NCBI using antiSMASH (Supplementary Tables S6 and S7). This revealed the NRPS region of *sphA* comprised of five modules, with a predicted sequence of Thr-Ser-Ser-X-X-TE (Supplementary Table S6), and confirming the previously described biosynthetic pathway of stephensiolides^17^. Moreover, due to the absence of epimerase enzymes (converts L-amino acids to D-amino acids) within the modules of the NRPS gene (*sphA*), the amino acids incorporated into the growing peptide chain possibly possess an L configuration (Supplementary Table S6). The NRPSpredictor2 web tool then predicted the specificity score of the adenylation-domain within each module of *sphA* with 100% for the first, second and third modules (Thr-Ser-Ser), 70–80% for the fourth module (Leu/Ile/Val/Phe) and 70% for the fifth module (Val/Ile/Leu). This corresponded to the MS^E^ data which indicated that the fourth and fifth modules of *sphA* are variable modules. Interestingly, nine of the 16 *Serratia* strains had a higher specificity score of 80% for the fourth module with Leu as the predicted amino acid. The BGC, herein described as *gcd*, encoding for NRPSs involved in glucosamine derivative biosynthesis was partially identified by Khilyas et al.^19^ within the genome of a pigmented *S.* *marcescens* SM6 strain. Of the 16 *Serratia* genomes that were screened for the *sphA* gene, only 3 genomes (18.8%) were found to contain the *gcd* comprised of one module (D-Val predicted with a specificity score of 90%) (Supplementary Table S8).

### Antibacterial activity of purified compounds

**Figure 4:**
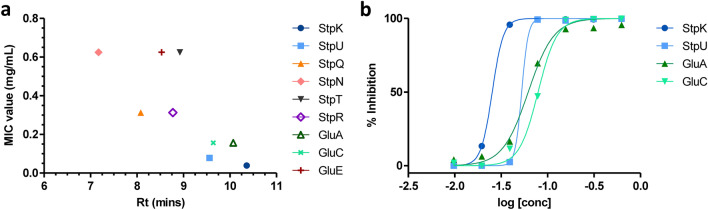
(**a**) The MIC values of the tested stephensiolides and glucosamine derivatives against *S. aureus* RN4220 plotted against their respective retention times. (**b**) The dose–response curves of the active StpU and StpK [*m/z* 682 (**12**) and 684 (**14**), respectively] and GluC and GluA [*m/z* 559 (**22**) and 585 (**26**), respectively].

Following structural elucidation, the antibacterial properties of the RP-HPLC purified compounds **3**, **8**, **9**, **11**, **12**, **14**, **21**, **22** and **26** were analysed against *S. aureus* RN4220 using a resazurin vitality assay^20^. Compounds **12** and **14** (i.e., StpU and StpK) exhibited promising antibacterial activity against *S.* *aureus*, with MIC values of 78 µg/mL and 39 µg/mL recorded, respectively. This was followed by compounds **22** and **26** (i.e., GluC and GluA), which both had MIC values of 156 µg/mL against *S.* *aureus*. Finally, weak or no activity was observed for compounds **3**, **8**, **9**, **11** and **21** (StpN, StpQ, StpR, StpT and GluE, respectively) against *S. aureus,* with MICs of ≥ 300 µg/mL. The activity of the various compounds was then compared to the hydrophobicity (the retention time of each compound; Fig. 4a), which indicated that the activity increased (lower MIC value) with an increase in the hydrophobicity (higher retention time on C_18_ matrix) of a compound. This interesting correlation with hydrophobicity could indicate that the bacterial cell membrane is a target of these compounds.

Dose–response curves were then generated for the active compounds, i.e., StpU, StpK, GluC and GluA, where both stephensiolides exhibited steeper activity slopes (differing Hill coefficient values)^21^ compared to the glucosamine derivatives (Fig. 4b), which could indicate different modes of action^22^. The stephensiolides exhibited a change in activity over a narrow concentration range indicating that a threshold concentration is needed for activity, such as needed for ion pore/channel formation^21^. Conversely, the glucosamine derivatives exhibited a flatter slope that could indicate detergent-like activity^21^. The IC_50_ values were derived from the sigmoidal curves (determined with GraphPad Prism), where StpU and StpK had IC_50_ values of 53 µg/mL and 25 µg/mL, respectively. In comparison, the GluC and GluA had IC_50_ values of 79 µg/mL and 61 µg/mL, respectively. To assess combined activity, checkerboard assays were conducted between GluA and StpU, GluA and StpK, GluC and StpK, as well as StpU and GluC. Only additive activity was observed between the selected glucosamine derivatives and stephensiolides against *S.* *aureus* (results not shown).

### Membrane permeability assay

**Figure 5:**
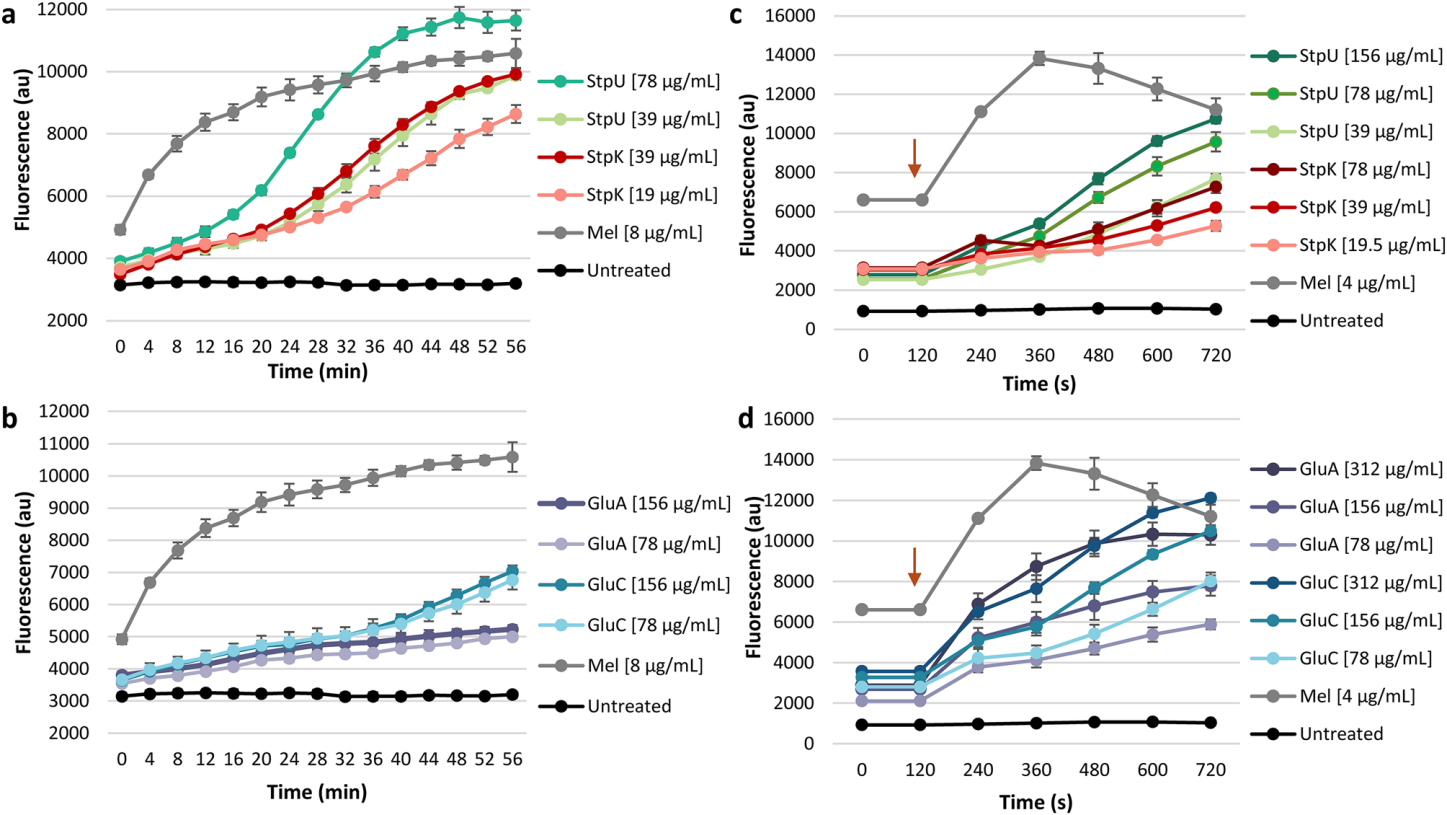
Membrane permeability of *S. aureus* RN4220 incubated with PI in the presence of ½MIC and MIC of (**a**) StpU and StpK [*m/z* 682 (**12**) and 684 (**14**), respectively] and (**b**) GluC and GluA [*m/z* 559 (**22**) and 585 (**26**), respectively]. Membrane potential measurements of exponentially growing *S.* *aureus* RN4220 cells incubated with DiSC_3_(5) in the presence of ½MIC, MIC and two-fold MIC of (**c**) StpU and StpK and (**d**) GluC and GluA. Melittin (Mel; ≥ 85%) served as a positive control and untreated cells served as negative control. au—arbitrary units. The red arrow indicates the first reading after cells were added.

To gain insight into whether stephensiolides and glucosamine derivatives cause increased membrane permeability by forming lesions within the cell membrane of *S. aureus*, membrane permeability was assessed by monitoring the influx of propidium iodide (PI) and subsequent staining of nucleic acids within the cell, resulting in a fluorescent signal^23,24^. Fluorescence was measured for 1 h at the MIC and ½MIC of compounds **12**, **14**, **22** and **26** (Fig. 5a and b).

Results indicated that StpU, StpK, GluC and GluA, at all concentrations analysed, induced an increase in PI fluorescence over time, indicating membrane permeabilisation and interaction of PI with nucleic acids. A two-fold increase in fluorescence was observed from the initial reading to within 28 and 36 min of exposure to the MIC of StpU and StpK (Fig. 5a), respectively. The incorporation of PI was additionally influenced by stephensiolide concentration, with a higher fluorescent signal observed for the higher concentrations at each time point. Although an increase in fluorescence was observed for GluC and GluA (Fig. 5b), less than a two-fold increase was obtained after 60 min. This result indicated that glucosamine derivatives do not cause major lesions to the membrane. The lytic control, melittin (≥ 85%; Sigma-Aldrich, St. Louis, USA), caused an expected increase in fluorescence signal, while no increase in fluorescence was observed for the negative control of *S. aureus* and PI (Fig. 5a and b).

### Membrane depolarisation assay

To gain insight into whether stephensiolides and glucosamine derivatives cause membrane depolarisation of *S. aureus*, membrane potential was assessed by monitoring the release of the voltage-sensitive dye 3,3′-dipropylthiadicarbocyanine iodide [DiSC_3_(5)]^25,26^. Fluorescence was thus measured for 12 min at two-fold MIC, MIC and ½MIC of compounds **12**, **14**, **22** and **26** (Fig. 5c and d).

Results indicated that StpU, StpK, GluC and GluA induced an increase in DiSC_3_(5) fluorescence at all concentrations tested, indicating rapid membrane depolarisation of the *S. aureus* cells. For the stephensiolides, an initial delay in membrane depolarisation was observed compared to the glucosamine derivatives. A two-fold increase in the fluorescent signal at MIC for StpK occurred after 720 s, while a two-fold increase in fluorescent signal at MIC for StpU occurred after 480 s (Fig. 5c). This result could be due to the formation of lesions in the form of pores or channels that are time-dependent on a self-assembly step. For the glucosamine derivatives, a two-fold increase (from the initial reading at 120 s) in fluorescent signal was observed within 360 s for the MIC of GluA, while a two-fold increase occurred within 480 s for GluC (Fig. 5d). This result correlated with a detergent-type permeabilisation where the loss of phospholipids from the membrane leads to membrane thinning and increased permeabilisation and depolarisation, but not large lesions. The depolarisation control of melittin cause rapid depolarisation within 240 s, while no increase in fluorescence was observed for the negative control of *S. aureus* and DiSC_3_(5).

## Discussion

Secondary metabolic profiling (using UPLC-MS^E^) and molecular networking analysis indicated that *S. marcescens* NP10 co-produced one known stephensiolide (K), sixteen new stephensiolides (L–Y), eight known glucosamine derivatives (A–E, H, J, K) and three new glucosamine derivatives (L–N). The new group of stephensiolides (L–Y) differed from those previously described, as NP10 exclusively produced stephensiolides that incorporated Leu/Ile into the fifth residue position, compared to a *Serratia* sp. isolated by Ganley et al.^17^ that predominantly produced stephensiolides (A–K) with Val at the fifth residue (except for StpK that had Leu/Ile incorporated into the fifth residue). This corresponded to genome mining and NRPSpredictor2 data which predicted that certain *Serratia* strains have a higher amino acid specificity (80%) of the adenylation-domain in module 4 (corresponding to residue 5) to incorporate Leu/Ile into the growing peptide compared to other strains (with 70% amino acid specificity) that were predicted to incorporate Val, Ile, Leu or Phe. It is thus hypothesised that NP10 has a higher specificity to Leu/Ile in module 4, resulting in a unique set of stephensiolides being produced. Moreover, genome mining and antiSMASH analysis of the 16 *Serratia* strains revealed that a significantly low proportion (18.8%) of these strains contained both glucosamine derivative and stephensiolide clusters, with secondary metabolic profiling analysis confirming the co-production of these two groups of compounds in this study for the first time.

The major compounds produced by NP10 were then purified using RP-HPLC and the antibacterial activity of the fractions was analysed. Results indicated that StpU, StpK, GluA and GluC exhibited activity against *S. aureus* with MICs ranging between 39 and 156 µg/mL. The StpK and StpU, with Ile/Leu in both residues 5 and 6 and a fatty acid chain length of C_12_ (with a double bond in the fatty acid chain for U), then exhibited the greatest activity (lowest MIC and IC_50_ values). The alteration of the peptide moiety in residue 5 and 6 potentially played a role in antimicrobial potency, where StpT (C_12:1_-Thr-Ser-Ser-Ile/Leu-Val; a more hydrophilic compound) with no activity observed against *S.* *aureus* differed from StpU (C_12:1_-Thr-Ser-Ser-Ile/Leu-Ile/Leu; a more hydrophobic compound) that had an MIC of 78 µg/mL against *S.* *aureus*. This suggests that replacing the final amino acid of Leu/Ile with Val (a less hydrophobic amino acid) may interfere with the antimicrobial activity. Moreover, StpN and StpR had the same peptide moiety as StpK (i.e., Thr-Ser-Ser-Ile/Leu-Ile/Leu); however, StpN and StpR had a shorter fatty acid chain (i.e., less hydrophobic) of C_8_ and C_10,_ respectively, compared to StpK with a fatty acid chain of C_12_ (i.e., more hydrophobic). This indicates that a shorter fatty acid chain length of C_10_ or C_8_ (resulting in reduced hydrophobicity) may have influenced the antibacterial activity of these stephensiolides and thus could potentially play a role in the mode of action of the compound^27,28^. When comparing the retention time (hydrophobicity) of these compounds, using UPLC analysis, to the antibacterial activity (MIC value), it was observed that the more hydrophobic lipopeptides (i.e., StpK and StpU) and glucosamines (GluA and GluC) that eluted later from the UPLC C_18_ column, exhibited greater antibacterial activity compared to the more hydrophilic compounds (StpN, StpQ, StpR, StpT and GluE). Previous studies have similarly found that more hydrophobic peptides (such as tyrocidines) and lipopeptides exhibited greater antibacterial potency, as hydrophobicity plays an important role in the initial interaction of these lipopeptides and peptides with the cell membrane and subsequent incorporation into the membrane^27,29^.

Dose–response curves were then generated to compare the potencies of the four active compounds (allows for analysis of the drugs' bactericidal properties). The sigmoidal curves indicated that the four compounds exhibited bactericidal activity, where a more lethal antibacterial action over a narrower concentration range was observed for stephensiolides due to a steeper activity slope in comparison to glucosamine derivatives with a more gradual slop^21^. The dose response curves suggest that at a threshold concentration of StpK or StpU, rapid killing of cells occurred. This indicates that a threshold concentration of lipopeptide on the cell membrane (due to lipopeptides accumulating on the surface of the membrane) is required for activity, such as the formation of pores within the cell membrane. This mode of action has been established for multiple lipopeptides^28,30^. In contrast, GluA and GluC exhibited a flatter slope that could indicate more detergent-like activity^21^.

The DiSC_3_(5) assay then revealed that the StpU and StpK caused membrane depolarisation of the target membrane within 8 and 12 min (at MIC), respectively. This was followed by the formation of lesions in the cell membrane, revealed by the PI assay, that affected the permeability within 28 and 36 min (to obtain two-fold increases) of exposure to MIC, and likely led to the leakage of other larger molecules and eventually cell death^30,31^. In comparison, GluA and GluC caused rapid membrane depolarisation within 6 and 8 min (at MIC), followed by minor membrane permeability of *S. aureus*. The depolarisation can either be caused by ion-conducting membrane pore formation by the glucosamine derivatives and increase ion-permeability or by acting as an ion carrier^26^. Furthermore, the dissipation of a transmembrane potential in many cases, can be the sole membrane mechanism of action and can inhibit or kill bacterial cells, or can contribute to the potency of the compound^26^. In this case, it seems that stephensiolides use a combination of membrane depolarisation and membrane permeability, which corresponds to known modes of action for lipopeptides^28,30^. In contrast, glucosamine derivatives primarily caused membrane depolarisation. However, time-kill assays need to be performed within 8 min of treatment to confirm if membrane depolarisation resulted in cell death of *S. aureus*.

Although the cytotoxicity of these compounds was not investigated, a previous study by Ganley et al.^17^ tested the cytotoxicity of a mixture of stephensiolides against the HepG2 cell line (human hepatocytes). The mixture was found to have an IC_50_ of 21 µg/mL against HepG2 cells; however, the cytotoxicity of purified (individual) stephensiolides, as well as glucosamine derivatives, remains unknown. Despite limited information regarding the cytotoxicity of these compounds, similar small lipopeptides with fatty acid chains of C_12_ and C_14_ have shown promise as potent antibacterial agents with low cytotoxicity^32^.

Finally, although the NP10 strain co-produced glucosamine derivatives and stephensiolides during secondary metabolism, it was observed that only an additive interaction occurred between these two types of compounds, against *S. aureus*. This may suggest that the strain may produce both compounds for alternative and/or various functions for the benefit of the producing strain. Lipopeptides have numerous functions that benefit the producing strain, including survival via antimicrobial activity, motility, adhesion and food acquisition, while glucosamine derivatives are currently only known to exhibit antibacterial activity^8,33^. These two groups of compounds may thus be co-produced to protect the producing strain against varying microbial antagonists in a natural environment.

## Conclusion

Molecular networking and MS^E^ analysis indicate that *S. marcescens* NP10 co-produces stephensiolides and glucosamine derivatives, of which the structures of sixteen new stephensiolides and three new glucosamine derivatives were described. Genome mining identified the BGCs involved in stephensiolide and glucosamine derivative production, where specificity scores of the adenylation-domains within each module in *sphA* (determined with NRPSpredictor2 within the antiSMASH web tool) corroborated MS^E^ data. Four compounds (i.e., StpK, StpU, GluA and GluC) had promising bactericidal activity against *S. aureus*; however, future research into the cytotoxicity of these compounds is still required. This suggests that *Serratia* spp. are a promising source of new bioactive antibacterial agents. Although the co-production of glucosamine derivatives and stephensiolides was observed, only additive interaction was observed between these two types of compounds against *S.* *aureus*. While further research into the mode of action is still required, this study is the first to report modes of membrane dependent activity of stephensiolides and glucosamine derivatives. The StpK and StpU caused membrane depolarisation followed by membrane permeabilisation of *S. aureus*, whilst GluA and GluC predominantly caused rapid membrane depolarisation.

## Methods

### Bacterial strains and growth conditions

The *S. marcescens* NP10 (non-pigmented) strain was previously isolated from a river water sample (Eerste River, Stellenbosch; GPS co-ordinates: -33.938998, 18.867430) and molecular typing confirmed the identity of the strain^7^. The *S. marcescens* NP10 strain was deposited in the South African Rhizobium Culture Collection (SARCC no. 3158) and is curated and accessible in the Water Resource Laboratory culture collection in the Department of Microbiology at Stellenbosch University (SU). The test microorganism used in the antimicrobial assay was the *S.* *aureus* RN4220 strain, and is curated and accessible in the BioPep™ Peptide Group culture collection in the Department of Biochemistry at SU. The bacterial strains were streaked from the glycerol stocks onto Nutrient agar (Merck, Johannesburg, South Africa) and were incubated at 37 °C for 18 to 24 h.

### Production and partial purification of secondary metabolites

The production of secondary metabolites by *S.* *marcescens* NP10 was performed as outlined in Clements et al.^6^. Briefly, triplicate 2 L baffled flasks containing 500 mL Peptone Glycerol (PG, pH 7.2 ± 0.2) broth were inoculated with a seed culture of NP10. The flasks were incubated at 30 °C for 120 h on an orbital shaker (MRCLAB, London, United Kingdom) at 120 rpm. After growth, cultures were centrifuged at 10,000 rpm for 20 min at 4 °C and the cell free supernatants were lyophilised. The lyophilised cell free supernatants were dissolved in 70% HPLC-grade MeCN (Romil, Darmstadt, Germany) in analytical quality H_2_O (prepared through a Millipore water filtration system) (*v/v*). The triplicate crude extracts were then pooled, lyophilised, analytically weighed and stored at − 20 °C until further use.

### Ultra-performance liquid chromatography coupled to tandem mass spectrometry

The pooled NP10 crude extract was analysed using UPLC-MS^E^ at the Liquid Chromatography Mass Spectrometry (LCMS) unit at the Central Analytical Facility (CAF, SU) as outlined in Clements-Decker et al.^4^. Briefly, the UPLC-MS^E^ was conducted on a Waters Synapt G2 high resolution mass spectrometer linked to an Acquity UPLC™ (Waters Corporation, Milford, USA). Three microlitres (1.00 mg/mL) was separated on an UPLC C_18_ reverse-phase analytical column (Acquity UPLC® HSS T3, 1.8 μm particle size, 2.1 × 150 mm, Waters Corporation, Dublin, Ireland). The UPLC was conducted with analytical quality H_2_O containing 0.1% (*v/v*) formic acid as solution A and MeCN containing 0.1% (*v/v*) formic acid as solution B at a flow rate of 0.300 mL/min as follows: 60% A from 0 to 0.5 min for loading, linear gradient from 40 to 95% (B) from 0.5 to 15 min and 95 to 40% (B) from 15 to 18 min. The positive mode ESI conditions included: 2.5 kV capillary voltage, 15 V cone voltage, 120 °C source temperature, desolvation temperature of 275 °C, 50 L/h cone gas flow, 650 L/h desolvation gas flow, and *m/z* range of 200 to 2000 in centroid mode.

High resolution collisionally induced dissociation (CID) analysis was conducted in the MS^E^ mode (MS/MS) during the UPLC-MS analysis and was monitored on a second MS channel. The collision energy of 6 eV was used for the function 1 and collision energy ramp from 20 to 70 eV at 1 s MS/MS scan time. Data was collected in the second mass analyser (MS2) through *m/z* range of 40 to 2000 in centroid mode. The remaining instrument settings were as described above. The UPLC-MS^E^ data was processed using MassLynx software version 4.2 (Waters Corporation, Milford, USA). The accurate masses and molecular formula of the detected compounds were used to search online databases, including Norine (https://bioinfo.lifl.fr/norine/) and PubChem (https://pubchem.ncbi.nlm.nih.gov/), of known natural products and an extensive literature search was conducted for the putative identification of the metabolites.

### Molecular networking

The molecular networking analysis was conducted as previously described by Clements et al.^8^ with minor modifications. Briefly, Reifycs Analysis Base File Converter was used to convert the Waters RAW file for NP10 into an Analysis Base File (ABF) format prior to data processing. Ion chromatogram extraction, alignment and peak deconvolution of the ABF converted file was then conducted using MS-DIAL software version 4.24. The aligned results were exported as a mascot generic format (mgf) file^34^. Thereafter, the mgf file was uploaded to the GNPS platform (http://gnps.ucsd.edu) and a molecular network was created using the workflow published by Wang et al.^35^. The following parameters were then used in the workflow: precursor ion mass tolerance was set to 0.03 Da, MS^E^ fragment ion tolerance of 0.02 Da, cosine score above 0.6 and minimum matched peaks of ten. The output of the molecular network was visualised using Cytoscape version 3.8.0.

### Reverse-phase high-performance liquid chromatography

The NP10 crude extract was subjected to RP-HPLC at the LCMS Central Analytical Facility Unit (CAF, Stellenbosch University), in order to obtain purified fractions as described by Clements-Decker et al.^4^. Briefly, RP-HPLC was conducted on an Acquity I-class UPLC™ system interfaced through a Xevo TQSmicro that is equipped with an electrospray ionisation source to a tandem quadrupole mass spectrometer (Waters Corporation, Milford, USA). The mass directed preparative RP-HPLC fractionation was performed on a XBridge Prep C_18_ OBD HPLC column (5 μm, 19 × 150 mm; Waters, Milford, MA, United States) using an injection volume of 500 to 1000 µL. The lyophilised NP10 crude extract was dissolved in 70% MeCN in analytical quality H_2_O (*v/v*) to a concentration of approximately 100 mg/mL. Liquid chromatography was conducted with analytical quality H_2_O containing 0.1% formic acid (Sigma-Aldrich, St. Louis, USA) as solution A and MeCN containing 0.1% formic acid (*v/v*) as solution B. The gradient was developed at a flow rate of 1.00 mL/min as follows: 50% B from 0 to 0.5 min for loading, linear gradient from 50 to 71% (B) from 0.5 to 20 min, 71 to 95% (B) from 20 to 21 min, and 95 to 50% (B) from 21.10 to 24 min. The chromatography of the lipopeptide analogues was followed by the detection of selected mass triggers (ranging from *m/z* 500 to 800). Fractions were automatically collected and lyophilised for antimicrobial testing. The following compounds were detected and purified (yield indicated in brackets): compounds **3** (0.068 mg), **8** (0.141 mg), **9** (0.556 mg), **11** (0.224 mg), **12** (1.563 mg), **14** (1.595 mg), **21** (0.373 mg), **22** (3.353 mg) and **26** (3.256 mg).

### ^1^H NMR analyses

Four selected lyophilised fractions purified via RP-HPLC (selected based on antimicrobial activity assays) were subjected to ^1^H NMR analyses. Lyophilised StpU and StpK were dissolved in 700 µL of CD_3_CN for ^1^H NMR and transferred to Norell Standard Series 5 mm NMR tubes (500 MHz) for comparison to Mai et al.^18^. Lyophilised GluA and GluC were dissolved in CD_3_OD and transferred to Norell Standard Series 5 mm NMR tubes (500 MHz) for comparison to Dwivedi et al.^14^. The ^1^H NMR data was acquired with a 9.4 T Varian Unity Inova spectrometer (operating at 600 MHz). The instrument is located at the Central Analytical Facility (CAF) at Stellenbosch University (SU). Spectra were referenced using solvent signals (CD_3_CN; δ_H_ 1.94 ppm and 2.130 ppm; CD_3_OD: δ_H_ 3.310 ppm and 4.870 ppm). All NMR spectra were processed using MestReNova version 6.0.2–5475.

StpU (**12**) C_34_H_59_N_5_O_9_; white solid; ^1^H NMR (600 MHz; CD_3_CN) is shown in Table 2; HR-ESIMS (*m/z*): [M + H]^+^ calcd. for C_34_H_60_N_5_O_9_, 682.4391; found, 682.4377.

StpK (**14**) C_34_H_61_N_5_O_9_; white solid; ^1^H NMR (600 MHz; CD_3_CN) is shown in Table 2; HR-ESIMS (*m/z*): [M + H]^+^ calcd. for C_34_H_62_N_5_O_9_, 684.4547; found, 684.4534.

GluC (**22**) C_29_H_54_N_2_O_8_; white solid; ^1^H NMR (600 MHz; CD_3_OD) is shown in Table 2; HR-ESIMS (*m/z*): [M + H]^+^ calcd. for C_29_H_55_N_2_O_8_, 559.3958; found, 559.3973.

GluA (**26**) C_31_H_56_N_2_O_8_; white solid; ^1^H NMR (600 MHz; CD_3_OD) is shown in Table 2; HR-ESIMS (*m/z*): [M + H]^+^ calcd. for C_31_H_57_N_2_O_8_, 585.4115; found, 585.4125.

### Genome mining for biosynthetic gene clusters

A previous study by Ganley et al.^17^ identified and described the biosynthetic pathway for stephensiolides. In contrast, the BGC responsible for glucosamine derivative synthesis was previously only detected by Khilyas et al.^19^, with no information regarding the gene or biosynthesis provided. In the current study, 17 *Serratia* genomes (Supplementary Table S7) were retrieved from the NCBI (https://www.ncbi.nlm.nih.gov/) and used for genome mining of the stephensiolide gene cluster (*sphA*) and the glucosamine derivative (*gcd*) gene cluster on antiSMASH software version 6.0. In addition, the NRPSpredictor2 (information obtain from within antiSMASH analysis) was then used to analyse the amino acid specificity of each adenylation-domain in the *sphA* and *gcd*^36^.

### Resazurin Broth microdilution assay

The RP-HPLC purified fractions were tested for antibacterial activity using a broth microdilution assay coupled with the metabolic dye, resazurin^20^ against *S. aureus* RN4220. The principle of the assay was based on the rate of reduction of the resazurin dye to resorufin, with survival of the cell equated to an increased conversion of this dye, while a decreased rate of conversion indicates impairment of cellular metabolism and was thus equated to cell death^20^. Briefly, the test strain was inoculated into 10 mL of cation adjusted-Mueller Hinton broth (Sigma-Aldrich) (CA-MHB containing 20–25 mg/mL calcium and 10–12.5 mg/mL magnesium) and incubated at 37 °C for 18 to 24 h. Following overnight incubation, the culture was diluted into the respective media and grown to mid-log phase to obtain an optical density (OD) at 0.3 (± 1.4 × 10^8^ cells mL^-1^) determined at 600 nm.

The RP-HPLC fractions were then diluted in MeCN to a concentration of 0.25 mg/mL. Fifty microlitres of each fraction was dispensed into the first row of the respective wells of a clear Greiner CELLSTAR® 96-well culture plate (Merck, Johannesburg, South Africa) containing 50 μL of analytical quality H_2_O, in triplicate. A microdilution was performed in the 96-well culture in 50 μL of sterile analytical quality H_2_O in each well and the plate was dried at 50 °C for 18 to 24 h to remove the solvent. Thereafter, 10 μL of target cells in mid-log phase at the adjusted OD was dispensed into each well, resulting in a final antimicrobial concentration range of each fraction from 9.8 to 625 µg/mL and incubated for 1 h at 37 °C. After 1 h incubation, 90 μL of phosphate buffered saline (PBS) was added to each well, followed by 10 μL of resazurin [prepared to a stock solution 0.3 mg/mL in PBS; Sigma-Aldrich (St Louis, MO, USA)]. The plate was then incubated at 37 °C for 1.5 h. Finally, fluorometric readings of the plate were conducted using a Tecan Spark 10 M Multimode Microplate Reader at 560 nm excitation wavelength and 590 nm emission wavelength. Sterile broth and the OD adjusted inoculum were included in the assay as a positive control, while sterile broth was included as a negative control. Tests were performed in triplicate, with independent duplicate experiments.

The percentage inhibition was calculated as described by van Rensburg et al.^20^. The MIC was considered the concentration that resulted in ≥ 90% inhibition of growth based on the fluorometric readings. The MIC vs retention time was plotted using Graphpad Prism version 3.01 for Windows [GraphPad Software, San Diego, USA (www.graphpad.com)]. Dose response curves (nonlinear regression) were additionally processed for the most active compounds (i.e., StpK, StpU, GluA and GluC) using Graphpad Prism version 5 as described by Rautenbach et al.^21^, and IC_50_ values for these four compounds were deduced from the best-fit curves.

### Checkerboard assay

A resazurin drugs combination microtiter plate assay was conducted to determine synergism between selected glucosamine derivatives and stephensiolides as outlined by Caleffi-Ferracioli et al.^37^ with minor modifications. Briefly, the *S. aureus* RN4220 strain was prepared in CA-MHB as described in the “Resazurin Broth Microdilution Assay” section and grown to mid-log phase to obtain an OD_600_ of 0.3 (± 1.4 × 10^8^ cells/mL). The RP-HPLC fractions (StpK, StpU, GluA and GluC; selected based on MICs) were serially diluted prior to addition a clear Greiner CELLSTAR® 96-well plate. The compounds were added in combination to the wells (i.e., GluA and StpU, GluA and StpK, GluC and StpK, as well as StpU and GluC) in triplicate, were after the solvent was removed (as described above). Thereafter, OD adjusted cells, PBS and resazurin were added to the wells and fluorometric readings were measured as described in the “Resazurin Broth Microdilution Assay” section. Sterile broth and the OD adjusted inoculum were included as a positive control, while sterile broth was included as a negative control. The fractional inhibitory concentration index (FICI) and synergistic effects were then determined using the following equation:

FICI = (C_A+B_/C_A_) + (C_B+A_/C_B_), where C_A_ and C_B_ are the MICs of the antibacterial compounds alone, and C_A+B_ and C_B+A_ are the MICs of the antibacterial compounds in combination. A FICI of ≤ 0.5 was interpreted as synergistic, a FICI between 0.5 and 4.0 as indifferent, and a FICI of ≥ 4.0 as antagonistic^37,38^.

### Membrane permeability assay: propidium iodide uptake

The membrane permeability capabilities of GluA, GluC, StpK and StpU on *S. aureus* RN4220 was determined using propidium iodide (PI) (Sigma-Aldrich; St. Louis, MO, USA)^23^. This fluorescent dye can only enter bacterial cells through membrane lesions that are big enough to allow the large molecule (PI has a molecular mass of 668.087 g/mol) to pass through, thus providing an indication of changes in membrane permeability^24^. Briefly, black Greiner CELLSTAR 96-well plates (Merck, Johannesburg, South Africa) were prepared with the selected four compounds at MIC and ½MIC and dried as described in the “Resazurin Broth Microdilution Assay” section. The *S. aureus* RN4220 strain was prepared in CA-MHB and grown to mid-log phase (OD_600_ of 0.5–0.6) and diluted to OD_600_ of 0.3. Propidium iodide (stock concentration of 1 mg/mL in water) was added to the cell suspension to a final concentration of 10 µg/mL and cells were incubated in the dark at room temperature for 10 min. Thereafter, 100 µL of OD adjusted PI cells in mid-log phase was dispensed into each well. Sterile broth and the OD adjusted inoculum with PI were included as a positive control, while sterile broth with PI was included as a negative control. Melittin served as a positive lytic control. Fluorometric readings of the plate were conducted in 2 min intervals over 60 min (readings began 120 s after cells were added to the compounds) using a Tecan Spark at 535 nm excitation wavelength and 617 nm emission wavelength.

### Membrane depolarisation assay: DiSC_3_(5)

The membrane depolarisation capabilities (ability to disrupt membrane potential) of GluA, GluC, StpK and StpU against *S.* *aureus* RN4220 was determined using the voltage-sensitive fluorescent probe, 3,3'-dipropylthiadicarbocyanine iodide [DiSC_3_(5)]^25,39^. Due to the cationic charge and hydrophobic nature of DiSC_3_(5), the fluorescent dye can penetrate and accumulate in lipid bilayers until Nernstian equilibrium is reached, resulting in the quenching of the fluorescent signal^26^. Once depolarisation occurs (i.e., changes in ion permeability and dissipating transmembrane potential), the dye is rapidly released into the medium and subsequently results in the dequenching of the fluorescent signal^26^. Briefly, black 96 well plates were prepared with the selected four compounds at two-fold MIC, MIC and ½MIC as described in the “Resazurin Broth Microdilution Assay” section. The *S. aureus* RN4220 strain was prepared in CA-MHB and grown to mid-log phase to obtain an OD_600_ of 0.5–0.6. Cells were harvested by centrifugation at 3000 rpm for 10 min and resuspended in HEPES buffer (5 mM, pH 7.4, containing 20 mM glucose; 100 mM KCl) to OD_600_ of 0.05. Thereafter, DiSC_3_(5) (stock concentration of 400 µM in 100% DMSO) was added to the cell suspension to a final concentration of 0.4 µM and cells were incubated in the dark at room temperature for 60 min. Cell suspensions (100 µL) were then dispensed into the wells of the 96 well plate containing the compounds. Sterile broth and the OD adjusted inoculum with DiSC_3_(5) were included as a non-fluorescent control, sterile broth with DiSC_3_(5) was included as a sterility control, and melittin was included as membrane depolarisation control. Fluorometric readings of the plate were conducted in 2 min intervals over 12 min (readings began 120 s after cells were added to the compounds) using a Tecan Spark at 622 nm excitation wavelength and 670 nm emission wavelength.

## Supplementary Information

Supplementary Information.

## Data Availability

All data generated or analysed during this study are included in this published article (and its Supplementary Information files). The genome sequence data of various *Serratia* strains available on the National Center for Biotechnology Information database (Accession numbers: LT575490.1; CP026383.1 AP013063.1; CP012685.1; JSFB01000001.1; CP012639.1; CP041134.1; CP011642.1; CP018923.1; AP024847.1; CP018924.1; CP033504.1; CP041130.1; CP041123.1; CP041125.1 and CP053927.1) was used for genome mining in this study. The *Serratia marcescens* NP10 strain is accessible from the South African Rhizobium Culture Collection (SARCC No. 3158).
