# Supplementary material for: Secondary metabolic profiling of Serratia marcescens NP10 reveals new stephensiolides and glucosamine derivatives with bacterial membrane activity
Source: Sci Rep. 2023 Feb 9;13:2360. doi: 10.1038/s41598-023-28502-6 (PMC9911388; doi:10.1038/s41598-023-28502-6)
Supplement: Supplementary file 1 — Supplementary Information. [file 41598_2023_28502_MOESM1_ESM.docx]

**Supplementary information**

**Secondary Metabolic Profiling of *Serratia marcescens* NP10 Reveals New Stephensiolides and Glucosamine Derivatives with Bacterial** **Membrane Activity**

Tanya Clements-Decker^1^, Marina Rautenbach^2^, Wilma Van Rensburg^2^, Sehaam Khan^1^, Marietjie Stander^2^ and Wesaal Khan^3*^

^1^Faculty of Health Sciences, University of Johannesburg, PO Box 17011, Doornfontein, 2028, South Africa

^2^Department of Biochemistry, Faculty of Science, Stellenbosch University, Private Bag X1, Stellenbosch, 7602, South Africa

^3^Department of Microbiology, Faculty of Science, Stellenbosch University, Private Bag X1, Stellenbosch, 7602, South Africa

Short title: Structure and membrane activity of new stephensiolides and glucosamine derivatives

*Corresponding Author - Wesaal Khan; Tel: +27 (21) 808 5804; E-mail: [wesaal@sun.ac.za](mailto:wesaal@sun.ac.za)

**Table of contents:**

[Table S1. Summary of the chemical parameters and proposed structure of glucosamine derivatives detected in the *S. marcescens* NP10 crude extract identified using proposed from the accurate *M_r_*, elemental composition and UPLC-MS^E^. Sequences in bold font were elucidated/confirmed with ^1^H NMR. 3](#_Toc118210041)

[Table S2. Summary of the compounds in the NP10 crude extract that were identified using UPLC-ESI-MS analysis. 4](#_Toc118210042)

[Table S3. The UPLC-MS^E^ analysis of the stephensiolides observed in the *Serratia marcescens* NP10 extract. Peptide fragments were annotated according to Roepstorff and Fohlman [5] nomenclature. 5](#_Toc118210043)

[Fig. S1. The ^1^H NMR spectra for (A) stephensiolide K (*m/z* 684.4534 [M+H]^+^) and (B) stephensiolide U (*m/z* 682.4377 [M+H]^+^) in CD_3_CN, as well as (C) glucosamine derivative A (*m/z* 585.4125 [M+H]^+^) and (D) glucosamine derivative C (*m/z* 559.3973 [M+H]^+^) in CD_3_OD. 7](#_Toc118210044)

[Fig. S2. The (A) MS^2^-fragmentation spectra (B) and structure of representative new compound ions [M+H]^+^ at *m/z* 658.4034 (stephensiolide O), 698.4340 (stephensiolide S), and 710.4691 (stephensiolide X). 8](#_Toc118210045)

[Table S4. Summary of the previously detected stephensiolides and the newly described analogues. 9](#_Toc118210046)

[Table S5. The UPLC-MS^E^ analysis of the glucosamine derivatives observed in the *Serratia marcescens* NP10 extract. 10](#_Toc118210047)

[Fig. S3. The MS^2^-fragmentation spectra and structures of the new compound ions [M+H]^+^ at *m/z* (A) 529.3513 (glucosamine derivative M), (B) 531.3650 (glucosamine derivative L) and (C) 601.4943 (glucosamine derivative N). 11](#_Toc118210048)

[Table S6. *Serratia* strains with a *sphA* (stephensiolide gene cluster) PKS-NRPS system and the NRPS region predicted by antiSMASH and NRPSpredictor2. 12](#_Toc118210049)

[Table S7. Summary of the PKS enzymes of the *sphA* biosynthetic gene cluster detected in the genomes of 16 *Serratia* strains using anti-SMASH software. 13](#_Toc118210050)

[Table S8. *Serratia* strains with a *gcd* (glucosamine derivative NRPS gene cluster) gene, additional biosynthetic genes and transport proteins predicted by antiSMASH. 14](#_Toc118210051)

# Table S1. Summary of the chemical parameters and proposed structure of glucosamine derivatives detected in the *S. marcescens* NP10 crude extract identified using proposed from the accurate *M_r_*, elemental composition and UPLC-MS^E^. Sequences in bold font were elucidated/confirmed with ^1^H NMR.

| **No.** | **R_t_ (min)** | **^a^ Abun-dance** | **^b^ Experimental**  **[M+H]^+^** | **^c^ Theoretical [M+H]^+^** | **^d^ PPM error** | **^e^ Proposed elementary composition** | **Proposed residue in glucosamine derivative, between residue 1 and 4** | | | | **Compound name** | **Reference** |
| --- | --- | --- | --- | --- | --- | --- | --- | --- | --- | --- | --- | --- |
| **18** | 7.13 | 0.3 | 529.3513 | 529.3489 | 5 | C_27_H_49_N_2_O_8_ | C_12:1_ | Val | Glucose | Butyric acid | *Glucosamine derivative M | N/A |
| **19** | 7.92 | 1.0 | 531.3650 | 531.3645 | 1 | C_27_H_51_N_2_O_8_ | C_12_ | Val | Glucose | Butyric acid | *Glucosamine derivative L | N/A |
| **20** | 7.85 | 2.5 | 575.3909 | 575.3907 | 0 | C_29_H_54_N_2_O_9_ | C_14:OH_ | Val | Glucose | Butyric acid | Glucosamine derivative D | [1] |
| **21** | 8.53 | 4.1 | 557.3804 | 557.3802 | 0 | C_29_H_52_N_2_O_8_ | C_14:1_ | Val | Glucose | Butyric acid | Glucosamine derivative E | [1] |
| **22** | 9.64 | 7.2 | 559.3973 | 559.3958 | 3 | C_29_H_54_N_2_O_8_ | **C_14_** | **Val** | **Glucose** | **Butyric acid** | Glucosamine derivative C | [1; 2] |
| **23** | 10.48 | 0.9 | 573.4144 | 573.4115 | 5 | C_30_H_56_N_2_O_8_ | C_15_ | Val | Glucose | Butyric acid | Glucosamine derivative B | [1; 2] |
| **24** | 8.34 | 0.5 | 601.4042 | 601.4064 | 4 | C_31_H_56_N_2_O_9_ | C_16:OH_ | Val | Glucose | Butyric acid | *Glucosamine derivative N | N/A |
| **25** | 9.82 | 0.6 | 583.3960 | 583.3958 | 0 | C_31_H_54_N_2_O_8_ | C_16:2_ | Val | Glucose | Butyric acid | Glucosamine derivative H | [1] |
| **26** | 10.07 | 5.2 | 585.4125 | 585.4115 | 2 | C_31_H_56_N_2_O_8_ | **C_16:1_** | **Val** | **Glucose** | **Butyric acid** | Glucosamine derivative A | [1; 2] |
| **27** | 10.99 | 0.2 | 627.4238 | 627.4220 | 3 | C_33_H_59_N_2_O_9_ | C_16:1_ | Val | Glucose | Oxo-hexanoic acid | Glucosamine derivative J | [1] |
| **28** | 11.26 | 2.8 | 587.4270 | 587.4271 | 0 | C_31_H_58_N_2_O_8_ | C_16_ | Val | Glucose | Butyric acid | Glucosamine derivative K | [1] |

^a^ Abundance was calculated from the UPLC-MS analyses of extracts. ^b^ Experimental protonated *M_r_* of a compound in the selected extract was calculated using the Time-of-Flight (TOF) transform function in the MassLynx 4.2 software package. ^c^ Theoretical protonated *M_r_* of compound was calculated using *de novo* *M_r_* calculation and verification with ChemDraw Ultra 12.0 software package. ^d^ Mass error in ppm = $\left( \frac{\mathrm{Theoretical}M_{r} - \mathrm{Experimental}M_{r}}{\mathrm{Theoretical}M_{r}} \right)\times{10}^{6}$. ^e^ Theoretical molecular formula of compound was calculated using ChemDraw Ultra 12.0 software package and experimental molecular formula was confirmed using the MassLynx 4.2 software package. No., Compound number; R_t_, Retention time. * Novel analogue.

|  |
| --- |

# Table S2. Summary of the compounds in the NP10 crude extract that were identified using UPLC-ESI-MS analysis.

| **No.** | **R_t_ (min)** | **^a^ Experimental**  ***m/z***  **[M+H]^+^** | **^a^ Experimental**  ***m/z***  **[M+Na]^+^** | **^a^ Experimental**  ***m/z***  **[M+K]^+^** | **^a^ Experimental**  ***m/z***  **[2M+H]^+^** | **^a^ Experimental**  ***m/z***  **[2M+Na]^+^** | **^a^ Experimental**  ***m/z***  **[2M+K]^+^** | **^b^ Calculated elementary composition** |
| --- | --- | --- | --- | --- | --- | --- | --- | --- |
| **1** | 5.46 | 644.3851 | 666.3658 | 682.3417 | 1287.7567 | 1309.7491 | nd | C_30_H_54_N_5_O_10_ |
| **2** | 6.45 | 614.3760 | 636.3600 | 652.3317 | 1227.7397 | 1249.7382 | 1265.6959 | C_29_H_51_N_5_O_9_ |
| **3** | 7.17 | 628.3920 | 650.3782 | 666.3458 | 1255.7764 | 1277.7523 | 1293.7316 | C_30_H_53_N_5_O_9_ |
| **4** | 4.95 | 690.4240 | 712.3926 | 728.3898 | 1379.8673 | nd | 1417.7510 | C_32_H_60_N_5_O_11_ |
| **5** | 6.22 | 674.4318 | 696.4124 | 712.3911 | 1347.8777 | 1369.8375 | 1385.7938 | C_32_H_60_N_5_O_10_ |
| **6** | 6.28 | 658.4034 | 680.3853 | 696.3577 | 1315.7957 | 1337.8074 | 1353.7539 | C_31_H_56_N_5_O_10_ |
| **7** | 7.00 | 672.4190 | 694.3946 | 710.3707 | 1343.8263 | 1365.8063 | 1381.7776 | C_32_H_58_N_5_O_10_ |
| **8** | 8.09 | 642.4078 | 664.3897 | 680.3612 | 1283.8014 | 1305.7843 | 1321.7589 | C_31_H_55_N_5_O_9_ |
| **9** | 8.79 | 656.4233 | 678.4052 | 694.3789 | 1311.8345 | 1333.8182 | 1349.7913 | C_32_H_57_N_5_O_9_ |
| **10** | 8.01 | 698.4340 | 720.4149 | 736.3934 | 1395.8687 | 1417.8402 | 1433.8317 | C_34_H_60_N_5_O_10_ |
| **11** | 8.92 | 668.4225 | 690.4044 | 706.3737 | 1335.8351 | 1357.8016 | 1373.7878 | C_33_H_57_N_5_O_9_ |
| **12** | 9.57 | 682.4377 | 704.4229 | 720.3990 | 1363.8674 | 1385.8517 | 1401.8282 | C_34_H_59_N_5_O_9_ |
| **13** | 9.74 | 670.4399 | 692.4230 | 708.3928 | 1339.8677 | 1361.8208 | 1377.8235 | C_33_H_59_N_5_O_9_ |
| **14** | 10.37 | 684.4534 | 706.4345 | 722.4064 | 1367.8958 | 1389.8864 | 1405.8529 | C_34_H_61_N_5_O_9_ |
| **15** | 10.31 | 696.4548 | 718.4328 | 734.4086 | 1391.8959 | 1413.8712 | 1429.8221 | C_35_H_61_N_5_O_9_ |
| **16** | 10.87 | 710.4691 | 732.4460 | 748.4274 | 1419.9178 | 1441.9131 | 1457.8433 | C_36_H_64_N_5_O_9_ |
| **17** | 11.88 | 712.4857 | 734.4691 | 750.4456 | 1423.9520 | 1445.9097 | 1461.9019 | C_36_H_66_N_5_O_9_ |
| **18** | 7.13 | 529.3513 | 551.3309 | 567.3033 | 1057.6849 | 1079.6805 | 1095.6544 | C_27_H_49_N_2_O_8_ |
| **19** | 7.92 | 531.3633 | 553.3473 | 569.3162 | 1061.7124 | 1083.6992 | 1099.6591 | C_27_H_51_N_2_O_8_ |
| **20** | 7.85 | 575.3891 | 597.3726 | 613.3455 | 1149.7727 | 1171.7496 | 1187.7242 | C_29_H_54_N_2_O_9_ |
| **21** | 8.53 | 557.3797 | 579.3650 | 595.3353 | 1113.7520 | 1135.7351 | 1151.6985 | C_29_H_52_N_2_O_8_ |
| **22** | 9.64 | 559.3950 | 581.3781 | 597.3550 | 1117.7834 | 1139.7682 | 1155.7441 | C_29_H_54_N_2_O_8_ |
| **23** | 10.48 | 573.4102 | 595.3553 | 611.3688 | 1145.8140 | 1167.7899 | 1183.7704 | C_30_H_56_N_2_O_8_ |
| **24** | 8.34 | 601.4055 | 623.3884 | 639.3643 | 1201.7990 | 1223.8026 | 1239.7683 | C_31_H_57_N_2_O_9_ |
| **25** | 9.82 | 583.3930 | 605.3688 | 621.3516 | 1165.7960 | 1187.7560 | 1203.6909 | C_31_H_54_N_2_O_8_ |
| **26** | 10.07 | 585.4112 | 607.3939 | 623.3663 | 1169.8125 | 1191.7947 | 1207.7694 | C_31_H_56_N_2_O_8_ |
| **27** | 10.99 | 627.4189 | 649.4003 | 665.3692 | 1253.8462 | 1275.8293 | nd | C_33_H_59_N_2_O_9_ |
| **28** | 11.26 | 587.4259 | 609.4085 | 625.3817 | 1173.8420 | 1195.8217 | 1211.7958 | C_31_H_58_N_2_O_8_ |

No., Compound number; R_t_, Retention time; 2M, MS stable non-covalent dimer.

^a^ Experimental protonated *M_r_* of a compound in the selected extract was calculated using the Time-of-Flight (TOF) transform function in the MassLynx 4.2 software package.

^b^ Theoretical elemental composition of compound was calculated using ChemDraw Ultra 12.0 software package and was confirmed using the MassLynx 4.2 software package.

# Table S3. The UPLC-MS^E^ analysis of the stephensiolides observed in the *Serratia marcescens* NP10 extract. Peptide fragments were annotated according to Roepstorff and Fohlman [5] nomenclature.

| **No** | **R_t_ (min)** | ^a^ **Experimental *m/z* [M+H]^+^** | **Residue 1** | **Residue 2** | **Residue 3** | **Residue 4** | **Residue 5** | **Residue 6** | **CID derived sequence, ring opening at ester bond between residue 1 and 6** |
| --- | --- | --- | --- | --- | --- | --- | --- | --- | --- |
|  |  |  | Experimental *m/z* of fragment (theoretical *m/z* of proposed fragment)  Proposed fragment identity containing residues from left to right, ring opening at ester bond between residue 1 and 6 | | | | | |  |
| 1 | 5.46 | 644.3851 | nd | 226.141 (226.144) | 313.179 (313.176) | 382.204 (382.198) | 513.282 (513.292) | 626.361 (626.376) | X_1_-Thr-Ser-Ser-(Ile/Leu)-(Ile/Leu) |
|  |  |  |  | b_2_-H, OH | b_3_-H, OH | b_4_-2(H, OH) | b_5_-H, OH | b_6_-H, OH |  |
| 2 | 6.45 | 614.3760 | 127.112 (127.112) | 210.151 (210.149) | 297.186 (297.181) | 384.215 (384.214) | 515.314 (515.308);  497.298 (497.298) | 596.361 (596.366) | C_8_H_14_O_2_-Thr-Ser-Ser-(Ile/Leu)-Val |
|  |  |  | b_1_ | b_2_-H, OH | b_3_-H, OH | b_4_-H, OH | b_5_; b_5_-H, OH | b_6_-H, OH |  |
| 3 | 7.17 | 628.3920 | nd | 228.160 (228.160);  210.150 (210.149) | 297.182 (297.181) | 384.213 (384.214) | 497.296 (497.298) | 610.384 (610.382) | X_1_-Thr-Ser-Ser-(Ile/Leu)-(Ile/Leu) |
|  |  |  |  | b_2_; b_2_-H, OH | b_3_-H, OH | b_4_-H, OH | b_5_; b_5_-H, OH | b_6_-H, OH |  |
| 4 | 4.95 | 690.4240 | nd | 272.186 (272.186);  254.174 (254.176) | 341.197 (341.208) | 428.227 (428.240) | 559.326 (559.334);  541.327 (541.324) | nd | X_1_-Thr-Ser-Ser-(Ile/Leu)-X_6_ |
|  |  |  |  | b_2_-H, OH; b_2_-2(H, OH) | b_3_-2(H, OH) | b_4_-2(H, OH) | b_5_-H, OH; b_5_-2(H, OH) |  |  |
| 5 | 6.22 | 674.4318 | nd | 256.190 (256.191) | 325.212 (325.213) | 430.246 (430.255) | 543.343 (543.339) | 656.424 (656.424) | X_1_-Thr-Ser-Ser-(Ile/Leu)-(Ile/Leu) |
|  |  |  |  | b_2_-H, OH | b_3_-2(H, OH) | b_4_-H, OH | b_5_-H, OH | b_6_-H, OH |  |
| 6 | 6.28 | 658.4034 | nd | 254.176 (254.176) | 341.203 (341.208) | 446.250 (446.250);  428.239 (428.240) | 559.339 (559.334);  541.322 (541.324) | 640.392 (640.392) | X_1_-Thr-Ser-Ser-(Ile/Leu)-Val |
|  |  |  |  | b_2_-H, OH | b_3_-H, OH | b_4_; b_4_-H, OH | b_5_; b_5_-H, OH | b_6_-H, OH |  |
| 7 | 7.00 | 672.4190 | nd | 254.175 (254.176) | 341.207 (341.208) | 446.252 (446.250);  428.237 (428.240) | 559.330 (559.334);  541.322 (541.324) | 654.405 (654.408) | X_1_-Thr-Ser-Ser-(Ile/Leu)-(Ile/Leu) |
|  |  |  |  | b_2_-H, OH | b_3_-H, OH | b_4_; b_4_-H, OH | b_5_; b_5_-H, OH | b_6_-H, OH |  |
| 8 | 8.09 | 642.4078 | nd | 256.191 (256.191);  238.181 (238.181) | 325.216 (325.213) | 412.248 (412.245) | 543.339 (543.339);  525.329 (525.329) | 614.414 (614.413);  624.397 (624.397) | X_1_-Thr-Ser-Ser-(Ile/Leu)-Val |
|  |  |  |  | b_2_; b_2_-H, OH | b_3_-H, OH | b_4_-H, OH | b_5_; b_5_-H, OH | a_6_; b_6_-H, OH |  |
| 9 | 8.79 | 656.4233 | nd | 256.194 (256.191);  238.181 (238.181) | 325.221 (325.213) | 412.245 (412.245) | 543.340 (543.339);  525.328 (525.329) | 638.409 (638.413) | X_1_-Thr-Ser-Ser-(Ile/Leu)-(Ile/Leu) |
|  |  |  |  | b_2_; b_2_-H, OH | b_3_-H, OH | b_4_-H, OH | b_5_; b_5_-H, OH | b_6_-H, OH |  |

No., Compound number; R_t_, Retention time; nd, not detected.

^a^ Experimental protonated *M_r_* of a compound in the selected extract was calculated using the Time-of-Flight (TOF) transform function in the MassLynx 4.2 software package.

**Table S3**. Continued.

| **No** | **R_t_ (min)** | ^a^ **Experimental *m/z* [M+H]^+^** | **Residue 1** | **Residue 2** | **Residue 3** | **Residue 4** | **Residue 5** | **Residue 6** | **CID derived sequence, ring opening at ester bond between residue 2 and 6** |
| --- | --- | --- | --- | --- | --- | --- | --- | --- | --- |
|  |  |  | Experimental *m/z* of fragment (theoretical *m/z* of proposed fragment)  Proposed fragment identity containing residues from left to right, ring opening at ester bond between residue 1 and 6 | | | | | |  |
| 10 | 8.01 | 698.4340 | nd | 280.192 (280.191) | 367.218 (367.223) | 454.246 (454.255) | 585.355 (585.350);  567.344 (567.339) | 680.426 (680.424) | X_1_-Thr-Ser-Ser-(Ile/Leu)-(Ile/Leu) |
|  |  |  |  | b_2_-H, OH | b_3_-H, OH | b_4_-H, OH | b_5_; b_5_-H, OH | b_6_-H, OH |  |
| 11 | 8.92 | 668.4225 | 163.150 (163.149) | 282.206 (282.207);  264.197 (264.196) | 351.230 (351.228) | 438.260 (438.261) | 551.344 (551.345) | 640.30 (640.429);  650.412 (650.413) | C_12_H_22_O_2_-Thr-Ser-Ser-(Ile/Leu)-Val |
|  |  |  | b_1_ | b_2_; b_2_-H, OH | b_3_-H, OH | b_4_-H, OH | b_5_-H, OH | a_6_; b_6_-H, OH |  |
| 12 | 9.57 | 682.4377 | nd | 254.213 (254.212);  282.210 (282.207);  264.197 (264.196) | 351.231 (351.228) | 438.259 (438.261) | 569.356 (569.355);  551.345 (551.345) | 664.428 (664.429) | X_1_-Thr-Ser-Ser-(Ile/Leu)-(Ile/Leu) |
|  |  |  |  | a_2_; b_2_; b_2_-H, OH | b_3_-H, OH | b_4_-H, OH | b_5_; b_5_-H, OH | b_6_-H, OH |  |
| 13 | 9.74 | 670.4399 | nd | 284.225 (284.223);  266.211 (266.212) | 353.248 (353.244) | 440.279 (440.276) | 553.362 (553.360) | 652.430 (652.429) | X_1_-Thr-Ser-Ser-(Ile/Leu)-Val |
|  |  |  |  | b_2_; b_2_-H, OH | b_3_-H, OH | b_4_-H, OH | b_5_-H, OH | b_6_-H, OH |  |
| 14 | 10.37 | 684.4534 | nd | 256.223 (256.228);  284.214 (284.223);  266.211 (266.212) | 353.244 (353.244) | 440.274 (440.276) | 571.368 (571.371);  553.358 (553.360) | 666.442 (666.444) | X_1_-Thr-Ser-Ser-(Ile/Leu)-(Ile/Leu) |
|  |  |  |  | a_2_; b_2_; b_2_-H, OH | b_3_-H, OH | b_4_-2(H, OH) | b_5_; b_5_-H, OH | b_6_-H, OH |  |
| 15 | 10.31 | 696.4548 | nd | 310.237 (310.238);  292.232 (292.228) | 379.265 (379.260) | 484.300 (484.302);  466.288 (466.292) | 597.386 (597.386);  579.380 (579.376) | 678.446 (678.444) | X_1_-Thr-Ser-Ser-(Ile/Leu)-Val |
|  |  |  |  | b_2_; b_2_-H, OH | b_3_-H, OH | b_4_; b_4_-H, OH | b_5_; b_5_-H, OH | b_6_-H, OH |  |
| 16 | 10.87 | 710.4691 | nd | 282.240 (282.243);  310.240 (310.238);  292.228 (292.228) | 379.261 (379.260) | 484.285 (484.302);  466.291 (466.292) | 597.386 (597.386);  579.373 (579.376) | 682.472 (682.476);  692.459 (692.460) | X_1_-Thr-Ser-Ser-(Ile/Leu)- (Ile/Leu) |
|  |  |  |  | a_2_; b_2_; b_2_-H, OH | b_3_-H, OH | b_4_; b_4_-H, OH | b_5_; b_5_-H, OH | a_6_; b_6_-H, OH |  |
| 17 | 11.88 | 712.4857 | nd | 294.243 (294.243) | 381.267 (381.275) | 450.293 (450.297) | 581.392 (581.392) | 694.465 (694.476) | X_1_-Thr-Ser-Ser-(Ile/Leu)-(Ile/Leu) |
|  |  |  |  | b_2_-H, OH | b_3_-H, OH | b_4_-2(H, OH) | b_5_-H, OH | b_6_-H, OH |  |

No., Compound number; R_t_, Retention time; nd, not detected.

^a^ Experimental protonated *M_r_* of a compound in the selected extract was calculated using the Time-of-Flight (TOF) transform function in the MassLynx 4.2 software package.

# **Fig. S1**. The ^1^H NMR spectra for (A) stephensiolide K (*m/z* 684.4534 [M+H]^+^) and (B) stephensiolide U (*m/z* 682.4377 [M+H]^+^) in CD_3_CN, as well as (C) glucosamine derivative A (*m/z* 585.4125 [M+H]^+^) and (D) glucosamine derivative C (*m/z* 559.3973 [M+H]^+^) in CD_3_OD.

# Fig. S2. The (A) MS^2^-fragmentation spectra (B) and structure of representative new compound ions [M+H]^+^ at *m/z* 658.4034 (stephensiolide O), 698.4340 (stephensiolide S), and 710.4691 (stephensiolide X).

# Table S4. Summary of the previously detected stephensiolides and the newly described analogues.

| **Name** | ***m/z***  **[M+H]^+^** | **Residue** | | | | | |
| --- | --- | --- | --- | --- | --- | --- | --- |
|  |  | **1** | **2** | **3** | **4** | **5** | **6** |
| **Known stephensiolides [3]** | | | | | | | |
| A | 600 | C_8_ | Thr | Ser | Ser | Val | Val |
| B | 614 | C_8_ | Thr | Ser | Ser | Val | Ile |
| C | 628 | C_10_ | Thr | Ser | Ser | Val | Val |
| D | 642 | C_10_ | Thr | Ser | Ser | Val | Ile |
| E | 654 | C_12:1_ | Thr | Ser | Ser | Val | Val |
| F | 668 | C_12:1_ | Thr | Ser | Ser | Val | Ile |
| G | 656 | C_12_ | Thr | Ser | Ser | Val | Val |
| H | 682 | C_13:1_ | Thr | Ser | Ser | Val | Ile |
| I | 670 | C_12_ | Thr | Ser | Ser | Val | Ile |
| J | 696 | C_14:1_ | Thr | Ser | Ser | Val | Ile |
| K | 684 | C_12_ | Thr | Ser | Ser | Ile | Ile |
| **New stephensiolides (this study)** | | | | | | | |
| L | 644 | C_8OH_ | Thr | Ser | Ser | Ile/Leu | Ile/Leu |
| M | 614 | C_8_ | Thr | Ser | Ser | Ile/Leu | Val |
| N | 628 | C_8_ | Thr | Ser | Ser | Ile/Leu | Ile/Leu |
| O | 658 | C_10OH_ | Thr | Ser | Ser | Ile/Leu | Val |
| P | 672 | C_10OH_ | Thr | Ser | Ser | Ile/Leu | Ile/Leu |
| Q | 642 | C_10_ | Thr | Ser | Ser | Ile/Leu | Val |
| R | 656 | C_10_ | Thr | Ser | Ser | Ile/Leu | Ile/Leu |
| S | 698 | C_12OH_ | Thr | Ser | Ser | Ile/Leu | Ile/Leu |
| T | 668 | C_12:1_ | Thr | Ser | Ser | Ile/Leu | Val |
| U | 682 | C_12:1_ | Thr | Ser | Ser | Ile | Ile |
| V | 670 | C_12_ | Thr | Ser | Ser | Ile/Leu | Val |
| W | 696 | C_14:1_ | Thr | Ser | Ser | Ile/Leu | Val |
| X | 710 | C_14:1_ | Thr | Ser | Ser | Ile/Leu | Ile/Leu |
| Y | 712 | C_14_ | Thr | Ser | Ser | Ile/Leu | Ile/Leu |
| Open-ring P | 690 | C_10OH_ | Thr | Ser | Ser | Ile/Leu | Ile+OH |
| Open-ring R | 674 | C_10_ | Thr | Ser | Ser | Ile/Leu | Ile+OH |

# Table S5. The UPLC-MS^E^ analysis of the glucosamine derivatives observed in the *Serratia marcescens* NP10 extract.

| **No.** | **R_t_ (min)** | **^a^ Experimental *m/z***  **[M+H]^+^** | **Experimental *m/z* of fragment (theoretical *m/z* of proposed fragment)**  Initial fragment at ester bond between valine and glucose | | | | | | | |
| --- | --- | --- | --- | --- | --- | --- | --- | --- | --- | --- |
|  |  |  | **1** | **2** | **3** | **4** | **5** | **6** | **7** | **8** |
|  |  |  | M – [Glu + BA] | [**1** + Na – H] | M – [Val + FA] | [**3** – H, OH] | [**4** – H, OH] | [**4** – C_4_H_6_O] | [**6** – H, OH] | [**5** – CH_2_O] |
| 18 | 7.13 | 529.3513 | 298.238 (298.238) | 320.220 (320.220) | 232.120  (232.119) | 214.109  (214.108) | 196.098  (196.097) | 144.066  (144.066) | 126.058  (126.056) | 166.087  (166.087) |
| 19 | 7.92 | 531.3650 | 300.254  (300.254) | 322.234 (322.236) | 232.121  (232.119) | 214.111  (214.108) | 196.100  (196.097) | 144.066  (144.066) | 126.051  (126.056) | 166.087  (166.087) |
| 20 | 7.85 | 575.3909 | 344.281 (344.280) | 366.257 (366.262) | 232.120  (232.119) | 214.110  (214.108) | 196.099  (196.097) | 144.066  (144.066) | 126.056  (126.056) | 166.087  (166.087) |
| 21 | 8.53 | 557.3804 | 326.270 (326.270) | 348.247 (348.252) | 232.119  (232.119) | 214.109  (214.108) | 196.098  (196.097) | 144.066  (144.066) | 126.056  (126.056) | 166.087  (166.087) |
| 22 | 9.64 | 559.3973 | 328.285 (328.285) | 350.266 (350.267) | 232.119  (232.119) | 214.109  (214.108) | 196.098  (196.097) | 144.067  (144.066) | 126.056  (126.056) | 166.088  (166.087) |
| 23 | 10.48 | 573.4144 | 342.299  (342.301) | 364.284 (364.283) | 232.118  (232.119) | 214.107  (214.108) | 196.095  (196.097) | 144.067  (144.066) | 126.056  (126.056) | 166.088  (166.087) |
| 24 | 8.34 | 601.4042 | 370.295 (370.296) | 392.280 (392.277) | 232.118  (232.119) | 214.109  (214.108) | 196.104  (196.097) | 144.068  (144.066) | 126.058  (126.056) | 166.086  (166.087) |
| 25 | 9.82 | 583.3960 | 352.284 (352.285) | 374.267 (374.267) | 232.119  (232.119) | 214.109  (214.108) | 196.101  (196.097) | 144.067  (144.066) | 126.057  (126.056) | 166.086  (166.087) |
| 26 | 10.07 | 585.4125 | 354.301 (354.301) | 376.284 (376.283) | 232.119  (232.119) | 214.108  (214.108) | 196.098  (196.097) | 144.067  (144.066) | 126.056  (126.056) | 166.087  (166.087) |
| 28 | 11.26 | 587.4270 | 356.318  (356.316) | 378.300  (378.298) | 232.120  (232.119) | 214.109  (214.108) | 196.098  (196.097) | 144.066  (144.066) | 126.058  (126.056) | 166.087  (166.087) |
| **No.** | **R_t_ (min)** | **Experimental *m/z***  **[M+H]^+^** | **1** | **2** | **3** | **4** | **5** | **6** |  |  |
|  |  |  | M – [Glu + OHA] | [**1** + Na – H] | M – [Val + FA] | [**3** – H, OH] | [**4** – C_4_H_6_O] | [**5** – H, OH] |  |  |
| 27 | 10.99 | 627.4238 | nd  (354.3008) | 376.282 (376.283) | 274.128  (274.129) | 256.118  (256.119) | 144.068  (144.066) | 126.050  (126.056) |  |  |

No., Compound number; R_t_, Retention time; nd, not detected; BA, butyric acid; OHA, oxo-hexanoic acid; FA, Fatty acid.

^a^ Experimental protonated *M_r_* of a compound in the selected extract was calculated using the Time-of-Flight (TOF) transform function in the MassLynx 4.2 software package.

# Fig. S3. The MS^2^-fragmentation spectra and structures of the new compound ions [M+H]^+^ at *m/z* (A) 529.3513 (glucosamine derivative M), (B) 531.3650 (glucosamine derivative L) and (C) 601.4943 (glucosamine derivative N).

# **Table S6**. *Serratia* strains with a *sphA* (stephensiolide gene cluster) PKS-NRPS system and the NRPS region predicted by antiSMASH and NRPSpredictor2.

| ***Serratia* strain** | **NCBI accession number** | ***sphA*** | **NRPS domains** | **AntiSMASH peptide sequence** | *** NRPSpredictor2 peptide sequence (score specificity %)** |
| --- | --- | --- | --- | --- | --- |
| *Serratia* sp. PWN146 | LT575490.1 | ✓ | (C-A-PCP)_1-5_-TE | Thr-Ser-Ser-Leu-X-TE | Thr(100%)-Ser(100%)-Ser(100%)-Leu(80%)- Val/Leu/Ile(70%) |
| *Serratia* sp. SSNIH1 | CP026383.1 | ✓ | (C-A-PCP)_1-5_-TE | Thr-Ser-Ser-Leu-X-TE | Thr(100%)-Ser(100%)-Ser(100%)-Leu(80%)-Val/Leu/Ile(70%) |
| *Serratia marcescens* SM39 | AP013063.1 | ✓ | (C-A-PCP)_1-5_-TE | Thr-Ser-Ser-Leu-X-TE | Thr(100%)-Ser(100%)-Ser(100%)-Leu(80%)- Val/Leu/Ile(70%) |
| *Serratia marcescens* SmUNAM836 | CP012685.1 | ✓ | (C-A-PCP)_1-5_-TE | Thr-Ser-Ser-Leu-X-TE | Thr(100%)-Ser(100%)-Ser(100%)-Leu(80%)- Val/Leu/Ile(70%) |
| *Serratia ureilytica* Lr5/4 LG59 | JSFB01000001.1 | ✓ | (C-A-PCP)_1-5_-TE | Thr-Ser-Ser-X-X-TE | Thr(100%)-Ser(100%)-Ser(100%)-Val/Leu/Ile/Phe(70%)- Val/Leu/Ile(70%) |
| *Serratia marcescens* RSC-14 | CP012639.1 | ✓ | (C-A-PCP)_1-5_-TE | Thr-Ser-Ser-X-X-TE | Thr(100%)-Ser(100%)-Ser(100%)-Val/Leu/Ile/Phe(70%)- Val/Leu/Ile(70%) |
| *Serratia marcescens* WVU-010 | CP041134.1 | ✓ | (C-A-PCP)_1-5_-TE | Thr-Ser-Ser-Leu-X-TE | Thr(100%)-Ser(100%)-Ser(100%)-Leu(80%)- Val/Leu/Ile(70%) |
| *Serratia marcescens* CAV1492 | CP011642.1 | ✓ | (C-A-PCP)_1-5_-TE | Thr-Ser-Ser-Leu-X-TE | Thr(100%)-Ser(100%)-Ser(100%)-Leu(80%)- Val/Leu/Ile(70%) |
| *Serratia marcescens* UMH9 | CP018923.1 | ✓ | (C-A-PCP)_1-5_-TE | Thr-Ser-Ser-Leu-X-TE | Thr(100%)-Ser(100%)-Ser(100%)-Leu(80%)- Val/Leu/Ile(70%) |
| *Serratia marcescens* 2020-O-9 DNA | AP024847.1 | ✓ | (C-A-PCP)_1-5_-TE | Thr-Ser-Ser-Leu-X-TE | Thr(100%)-Ser(100%)-Ser(100%)-Leu(80%)- Val/Leu/Ile(70%) |
| *Serratia marcescens* UMH2 | CP018924.1 | ✓ | (C-A-PCP)_1-5_-TE | Thr-Ser-Ser-X-X-TE | Thr(100%)-Ser(100%)-Ser(100%)-Val/Leu/Ile/Phe(70%)- Val/Leu/Ile(70%) |
| *Serratia* sp. LS-1 | CP033504.1 | ✓ | (C-A-PCP)_1-5_-TE | Thr-Ser-Ser-X-X-TE | Thr(100%)-Ser(100%)-Ser(100%)-Val/Leu/Ile/Phe(70%)- Val/Leu/Ile(70%) |
| *Serratia marcescens* WVU-007 | CP041130.1 | ✓ | (C-A-PCP)_1-5_-TE | Thr-Ser-Ser-Leu-X-TE | Thr(100%)-Ser(100%)-Ser(100%)-Leu(80%)- Val/Leu/Ile(70%) |
| *Serratia marcescens* WVU-002 | CP041123.1 | ✓ | (C-A-PCP)_1-5_-TE | Thr-Ser-Ser-Leu-X-TE | Thr(100%)-Ser(100%)-Ser(100%)-Leu(80%)- Val/Leu/Ile(70%) |
| *Serratia marcescens* WVU-004 | CP041125.1 | ✓ | (C-A-PCP)_1-5_-TE | Thr-Ser-Ser-Leu-X-TE | Thr(100%)-Ser(100%)-Ser(100%)-Leu(80%)- Val/Leu/Ile(70%) |
| *Serratia marcescens* 11/2010 | CP053927.1 | ✓ | (C-A-PCP)_1-5_-TE | Thr-Ser-Ser-Leu-X-TE | Thr(100%)-Ser(100%)-Ser(100%)-Leu(80%)-Val/Ile/Leu(70%) |

C, Condensation domain; A, Adenylation domain; PCP, Peptidyl carrier protein; TE, Thioesterase; X, variable amino acid prediction. *The Nearest Neighbour predictor for each query the specificity of the most similar sequence within the database of annotated A-domain sequences (based on Stachelhaus-code).

# **Table S7**. Summary of the PKS enzymes of the *sphA* biosynthetic gene cluster detected in the genomes of 16 *Serratia* strains using anti-SMASH software.

| ***Serratia* strain** | **PKS enzymes** | | | | | | | |
| --- | --- | --- | --- | --- | --- | --- | --- | --- |
|  | Enoylreductase  Alcohol dehydrogenase or Oxidoreductase | Acetyl-CoA carboxylase biotin carboxylase subunit | Glutathione S-transferase | Ketoreductase  3-oxoacyl-Acyl-Carrier-Protein reductase | Putative short chain dehydrogenase | Acetyltransferase  maltose O- acetyltransferase | Keto reductase | Aminotransferase |
| PWN146 | X | X | X |  | X | X |  | X |
| SSNIH1 | X | X | X |  | X | X |  | X |
| SM39 | X | X | X |  | X | X |  | X |
| SmUNAM836 | X | X | X |  | X | X |  | X |
| Lr5/4 LG59 | X | X | X |  | X | X |  | X |
| RSC-14 | X | X | X |  | X | X |  | X |
| WVU-010 | X | X | X |  | X | X |  | X |
| CAV1492 | X |  | X |  | X | X |  | X |
| UMH9 | X | X | X |  | X | X |  | X |
| 2020-O-9 DNA | X | X | X | X |  | X |  | X |
| UMH2 | X | X | X |  | X | X |  | X |
| LS-1 | X | X | X |  | X | X |  | X |
| WVU-007 | X | X | X | X |  | X |  | X |
| WVU-002 | X | X | X | X |  | X |  | X |
| WVU-004 | X | X | X | X |  | X |  | X |
| 11/2010 | X | X | X | X |  | X | X | X |

# Table S8. *Serratia* strains with a *gcd* (glucosamine derivative NRPS gene cluster) gene, additional biosynthetic genes and transport proteins predicted by antiSMASH.

| ***Serratia* strain** | **NCBI accession number** | ***gcd gene*** | **NRPS domains** | **AntiSMASH peptide sequence (NRPSpredictor2 specificity %)** | **Additional biosynthetic genes** | **Transport proteins** | | |
| --- | --- | --- | --- | --- | --- | --- | --- | --- |
|  |  |  |  |  | Hydrolase | MATE efflux family protein | Peptidase | RND family efflux transporter MFP subunit |
| *Serratia* sp. PWN146 | LT575490.1 | ✓ | (C-A-PCP-E) | D-Val (90%) | X | X | X | X |
| *Serratia* sp. SSNIH1 | CP026383.1 | ✓ | (C-A-PCP-E) | D-Val (90%) | X | X | X | X |
| *Serratia marcescens* CAV1492 | CP011642.1 | ✓ | (C-A-PCP-E) | D-Val (90%) | X | X | X | X |
| *S. marcescens* SM6  [4] | SDUW00000000 | ✓ | (C-A-PCP-E) | D-Val (90%) | X | X | X | X |

C, Condensation domain; A, Adenylation domain; PCP, Peptidyl carrier protein; E, Epimerase.

**References**

1. Clements, T., Rautenbach, M., Ndlovu, T., Khan, S. and Khan, W. (2021). A metabolomics and molecular networking approach to elucidate the structures of secondary metabolites produced by *Serratia marcescens* strains. *Front. Chem.* 9, 633870. doi: 10.3389/fchem.2021.633870
2. Dwivedi, D., Jansen, R., Molinari, G., Nimtz, M., Johri, B. N., and Wray, V. (2008). Antimycobacterial serratamolides and diacyl peptoglucosamine derivatives from *Serratia* sp. *J. Nat. Prod.* 71 (4), 637–641. doi:10.1021/np7007126
3. Ganley, J.G., Carr, G., Ioerger, T.R., Sacchettini, J.C., Clardy, J. and Derbyshire, E.R. (2018). Discovery of antimicrobial lipodepsipeptides produced by a *Serratia* sp. within mosquito microbiomes. *ChemBioChem* 19 (15), 1590-1594. doi: 10.1002/cbic.201800124
4. Khilyas, I.V., Tursunov, K.A., Shirshikova, T.V., Kamaletdinova, L.K., Matrosova, L.E., Desai, P.T., McClelland, M. and Bogomolnaya, L.M. (2019) Genome sequence of pigmented siderophore-producing strain *Serratia marcescens* SM6. *Microbiol. Resour. Announc.* *8*, e00247-19. doi: 10.1128/MRA.00247-19
5. Roepstorff, P. and Fohlman, J. (1984). Proposal for a common nomenclature for sequence ions in mass spectra of peptides. *Biomed. Mass Spectrom.* 11, 601-605. doi: 10.1002/bms.1200111109
